# DNA replication initiation factor RECQ4 possesses a role in antagonizing DNA replication initiation

**DOI:** 10.1038/s41467-023-36968-1

**Published:** 2023-03-04

**Authors:** Xiaohua Xu, Chou-Wei Chang, Min Li, Kenneth Omabe, Nhung Le, Yi-Hsuan Chen, Feng Liang, Yilun Liu

**Affiliations:** 1 Thermo Fisher Scientific, 5781 Van Allen Way, Carlsbad, CA 92008 USA; 2 Vesigen Therapeutics, 790 Memorial Drive, Suite 103, Cambridge, MA 02139 USA; 3 Department of Cancer Genetics and Epigenetics, Beckman Research Institute, City of Hope, Duarte, CA 91010-3000 USA; 4 Department of Computer Science, University of Southern California, Los Angeles, CA 90089 USA; 5 Neuroscience Graduate Program, University of Southern California, Los Angeles, CA 90089 USA

**Keywords:** Origin firing, Ubiquitylation, Origin firing

## Abstract

Deletion of the conserved C-terminus of the Rothmund-Thomson syndrome helicase RECQ4 is highly tumorigenic. However, while the RECQ4 N-terminus is known to facilitate DNA replication initiation, the function of its C-terminus remains unclear. Using an unbiased proteomic approach, we identify an interaction between the RECQ4 N-terminus and the anaphase-promoting complex/cyclosome (APC/C) on human chromatin. We further show that this interaction stabilizes APC/C co-activator CDH1 and enhances APC/C-dependent degradation of the replication inhibitor Geminin, allowing replication factors to accumulate on chromatin. In contrast, the function is blocked by the RECQ4 C-terminus, which binds to protein inhibitors of APC/C. A cancer-prone, C-terminal-deleted RECQ4 mutation increases origin firing frequency, accelerates G_1_/S transition, and supports abnormally high DNA content. Our study reveals a role of the human RECQ4 C-terminus in antagonizing its N-terminus, thereby suppressing replication initiation, and this suppression is impaired by oncogenic mutations.

## Abstract (web summary)

RECQ4 mutations contribute to multiple developmental diseases and tumorigenesis. Here the authors describe how a highly oncogenic RECQ4 mutation alters the control of DNA synthesis, leading to abnormal DNA content and cell growth.

## Introduction

Rothmund-Thomson syndrome (RTS), Baller-Gerald syndrome, and RAPADILINO are diseases linked to mutations in the human *RECQ4* gene, which encodes a member of the RECQ family of DNA helicases^1–4^. A conserved superfamily II (SFII) helicase domain is located at the center of the RECQ4 protein, flanked by amino (N) and carboxyl (C) termini containing unique sequences that are distinct from other members of the RECQ family. The RECQ4 N-terminus is essential for development, because its deletion leads to embryonic lethality in mice^5–7^. Further, it shares sequence homology with the essential DNA replication initiation factor Sld2 in yeast^8,9^. Consistent with a role in replication initiation, RECQ4 binds to replication origins, and the SLD2-like domain of the human RECQ4 N-terminus forms a cell cycle-dependent, chromatin-bound protein complex that contains core replication factors, including MCM10, MCM2-7 helicase, CDC45, and GINS^8–12^. The interactions of RECQ4 with these replication components are important for the assembly of active CDC45-MCM2-7-GINS replicative helicase on chromatin to initiate DNA synthesis^10,11^. Hence, it has been shown that artificial tethering of RECQ4 to replication origins is sufficient to initiate DNA replication^13^.

Deregulated DNA synthesis has been linked to human diseases including cancer^14–16^. Indeed, a subset of RECQ4 clinical mutations are tumorigenic^3^, making RECQ4 an ideal candidate to study the contribution of DNA synthesis dysfunction to tumorigenesis. One of the most commonly found RECQ4 clinical mutations, del(Ala420_Ala463), causes RAPADILINO and is associated with a significantly higher risk of lymphoma^3^. This mutation leads to an internal deletion (ID) of 44 amino acids within the N-terminus immediately upstream of the SFII helicase domain. These 44 residues within the ID motif are involved in protein–protein interactions; one of these interacting proteins is p32/HABP1, which negatively controls RECQ4 mitochondrial localization^17^. In addition to initiating DNA replication in the nucleus, RECQ4 also interacts with the mitochondrial replicative helicase TWINKLE and is targeted to the mitochondria for mitochondrial DNA (mtDNA) synthesis^17^. Hence, the ID mutation impairs RECQ4 interaction with p32 and results in accumulation of the mutant protein in the mitochondria, mtDNA overproduction, and mitochondrial dysfunction^17^. In addition to the ID mutation, the RECQ4 clinical mutation Q757X is also oncogenic^3^. Individuals who are homozygous or compound heterozygous for the Q757X mutation carry a 40% risk of developing osteosarcoma^3^. Q757X is a truncation mutation, in which the conserved C-terminus downstream of the SFII domain is deleted. Interestingly, a RECQ4 splicing mutation (R766X), which leads to a similar C-terminal truncation, has been observed frequently in the tumor registry^18^ and is considered oncogenic. Nonetheless, despite an important role in tumor suppression, the molecular function of the RECQ4 C-terminus remains unclear.

In this work, we report an unexpected role of the RECQ4 C-terminus in antagonizing the N-terminus, thereby reducing replication origin firing. Using an unbiased proteomic approach, we identify the anaphase-promoting complex/cyclosome (APC/C), an essential E3 ubiquitin ligase that drives cell cycle progression^19^, as a factor that interacts with the RECQ4 N-terminus on human chromatin. This interaction peaks at S phase and enhances APC/C-mediated degradation of the replication inhibitor Geminin, replisome assembly, and origin firing. However, this interaction is restricted by the RECQ4 C-terminus, which binds to the APC/C inhibitor protein EMI1. Consequently, cells carrying cancer-associated RECQ4 C-terminal truncation mutations missing the EMI1-interacting motif show accelerated G_1_/S transition and higher-than-normal DNA content, possibly due to DNA re-replication. Our work demonstrates that the RECQ4 C-terminus possesses a self-regulatory mechanism that prevents replication initiation by suppressing RECQ4 N-terminal interaction with APC/C. APC/C is a multi-protein complex, and *ANAPC1*, which encodes APC1, the largest subunit of APC/C, has been identified as the second RTS genetic risk factor^20^. Our study provides a functional link between the two RTS genes in DNA replication control.

## Results

### The RECQ4 C-terminus antagonizes DNA replication initiation

Consistent with the essential role of RECQ4 in the mammalian system^5^, our previous attempt to isolate viable RECQ4-null HEK293 cells using CRISPR technology was not successful^21^. Instead, because CRISPR-generated frameshift mutations are known to yield low levels of synthesized protein due to ribosomal slippage^22^, we isolated HEK293 cells that contained RECQ4 frameshift mutations in both alleles^21^. We referred to these RECQ4-CRISPR-edited cells as stable RECQ4 knockdown (KD) cells (Supplementary Fig. 1a), as they survived due to a reduced but detectable amount of RECQ4 on chromatin^21^. In the present study, we found that these RECQ4 KD cells showed an almost 2-fold increase in Geminin (Supplementary Fig. 1a), which blocks DNA replication initiation by inhibiting replication initiation factors^23^. Indeed, this increase was accompanied by a decrease in the replication initiation factors CDT1 and MCM7 (Supplementary Fig. 1a). As a result, RECQ4 KD cells proliferated slower (Supplementary Fig. 1b) and accumulated in G_1_ phase compared to wild-type (WT) HEK293 cells (Supplementary Fig. 1c). When we stably expressed a FLAG-tagged RECQ4 WT construct in RECQ4 KD cells at a similar level as the endogenous RECQ4 protein in the parental HEK293 cells (Supplementary Fig. 1d), the slow growth rate of the RECQ4 KD cells was restored to a level comparable to that of parental HEK293 cells (Supplementary Fig. 1e).

**Fig. 1:**
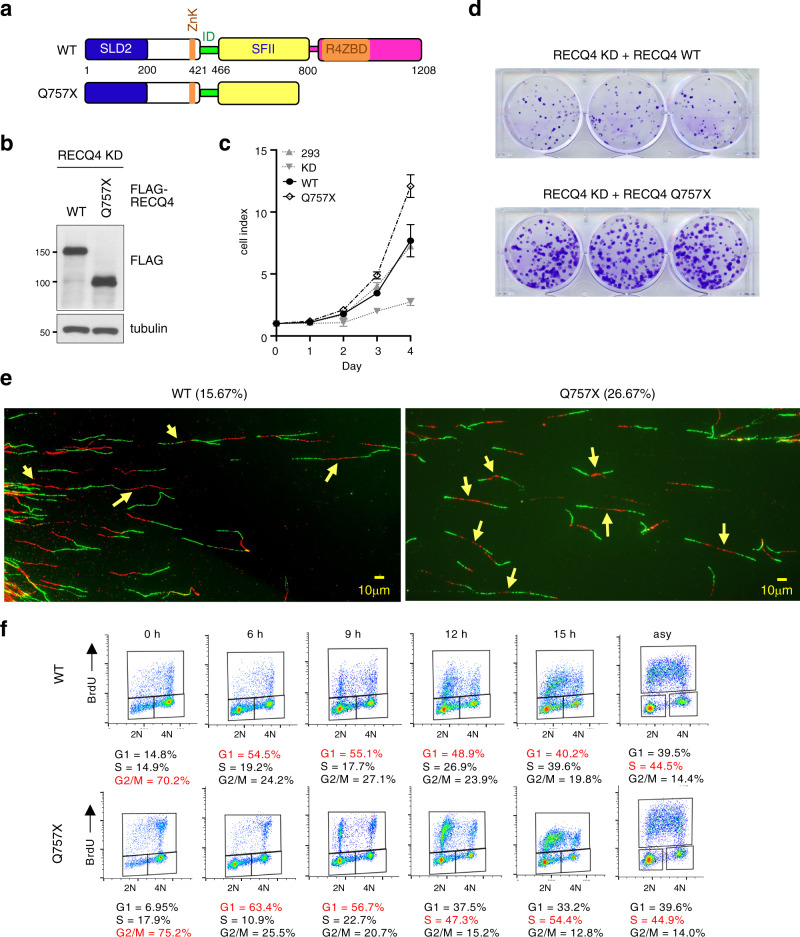
RECQ4 C-terminus antagonizes DNA replication initiation. **a** A schematic diagram of human RECQ4 wild type (WT, top) and Q757X mutant protein (bottom), including the SLD2, internal deletion (ID), superfamily helicase II (SFII), and two proposed zinc-binding motifs (ZnK and R4ZBD). Residues of the domain boundaries are shown. **b** Representative western blot analysis (anti-FLAG) for the presence of RECQ4 WT and Q757X mutant proteins in whole-cell extracts (WCE) prepared from RECQ4 knockdown (KD) HEK293 cells stably expressing FLAG-tagged RECQ4 WT or Q757X mutant proteins. Tubulin was used as a loading control. **c** Representative real-time cell growth assays to measure cell growth rate of HEK293 or RECQ4 KD cells with or without stable expression of FLAG-RECQ4 WT or Q757X mutant. Source data are provided as a Source data file. *n* = 3 (biologically independent samples). Data are presented as mean values ± SD. **d** Representative colony formation assay to measure growth of RECQ4 knockdown (KD) cells with stable expression of FLAG-RECQ4 WT (top) or Q757X mutant (bottom) at Day 14 post seeding. **e** Representative fluorescence images of DNA fibers isolated from RECQ4 WT (left) or Q757X mutant (right) expressing cells. DNA fibers exhibiting origin firing are indicated with yellow arrows. The percentage of labeled DNA exhibiting origin firing from at least 150 DNA fibers is shown. **f** Flow cytometry analysis of RECQ4 WT (top) or Q757X mutant (bottom) expressing cells synchronized at the indicated time points after release from nocodazole blocking (left panels) or asynchronized (asy; right panels). The cells were labeled with BrdU and co-stained for BrdU incorporation (Y-axis) and DNA content (propidium iodide [PI], X-axis). Percentages of cells in G_1_, S, and G_2_/M phases are shown, with the highest percentage in each time point indicated in red.

To determine the function of the RECQ4 C-terminus, we stably expressed a FLAG-tagged RECQ4 construct containing the cancer-associated Q757X mutation in RECQ4 KD cells (Fig. 1a). When FLAG-RECQ4 WT or Q757X mutant proteins were expressed at similar levels (Fig. 1b), RECQ4 Q757X mutant cells exhibited a faster growth rate than RECQ4 WT cells (Fig. 1c, d). Because RECQ4 is involved in initiating DNA replication^8–12^, we performed DNA fiber analysis using asynchronized RECQ4 WT and Q757X mutant cells and found that the frequency of origin firing per IdU/CIdU-positive DNA fiber was greater in Q757X mutant cells (26.67%) than in WT cells (15.67%) (Fig. 1e, yellow arrows). This increase in origin firing was not due to a higher percentage of the S-phase population in Q757X mutant cells compared to WT cells (Fig. 1f, right panels), suggesting that there was an increase in origin firing frequency per each Q757X mutant cell undergoing DNA synthesis. Increasing origin firing can shorten S phase^24^, explaining the faster growth rate in the RECQ4 Q757X mutant cells. Shortening S phase is expected to decrease the S-phase population in asynchronized cells, but the ratio between G_1_- and S-phase populations did not change in asynchronized Q757X mutant cells compared to asynchronized WT cells (Fig. 1f, right panels). Therefore, we tested the possibility that DNA replication was initiated earlier to shorten the G_1_ phase in Q757X mutant cells. To test this, we used nocodazole to synchronize RECQ4 WT and Q757X mutant cells to the G_2_/M phase. At 6 h post-nocodazole release, more than 50% of the RECQ4 WT and Q757X mutant cells had transitioned into G_1_ (Fig. 1f). However, by 12 h post-nocodazole release, a higher percentage of Q757X mutant cells (47.3%) were BrdU-positive compared to WT cells (26.9%; Fig. 1f), indicating more Q757X mutant cells had transitioned into S phase, which was maintained at 15 h post-nocodazole release (Fig. 1f), supporting our conclusion that G_1_ phase is shortened by the Q757X mutation.

Replication stress induced by hydroxyurea (HU) or camptothecin (CPT) activates additional replication origins that are normally dormant to help maintain DNA synthesis rate and increase cell survival^25–27^. To test if increased origin firing by the RECQ4 Q757X mutation relates to dormant origins, we assessed the sensitivity of RECQ4 WT or Q757X mutant cells to HU and CPT and found that both chemicals inhibited WT and Q757X mutant cell growth (Supplementary Fig. 2a). RECQ4 forms a chromatin-specific replication complex via the N-terminus to initiate DNA replication^10^. When we treated RECQ4 WT or Q757X mutant cells with ionizing radiation to induce DNA damage, both WT and Q757X replication complexes were reduced (Supplementary Fig. 2b). These results reveal a role for the RECQ4 C-terminus in suppressing replication origin firing, and suggest this function is not related to cellular response to replication stress.

### The RECQ4 C-terminus limits interactions with replication factors and APC/C

**Fig. 2:**
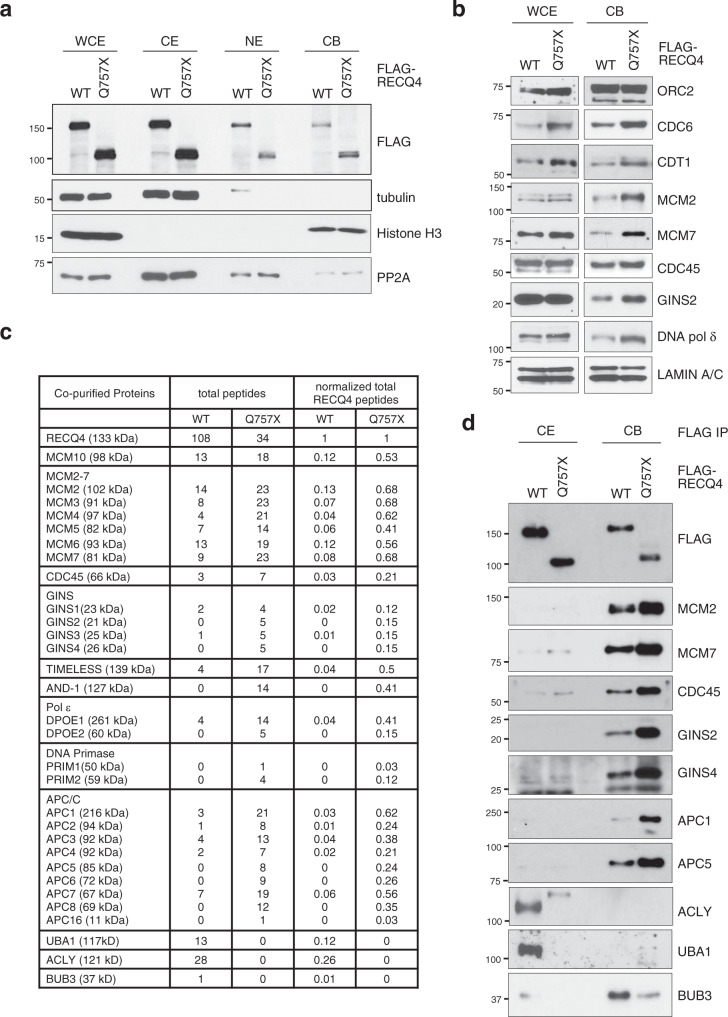
The RECQ4 C-terminus regulates protein–protein interactions. **a** Representative western blot analysis of the indicated proteins in whole-cell extracts (WCE) and cytoplasmic (CE), nucleoplasmic (NE), and chromatin-bound (CB) fractions prepared from RECQ4 knockdown (KD) HEK293 cells stably expressing FLAG-tagged RECQ4 wild type (WT) or Q757X mutant proteins. Tubulin, histone H3, and PP2A are loading and fractionation controls. **b** Representative western blot analysis of the indicated proteins in WCE and CB fractions prepared from RECQ4 WT and Q757X mutant expressing cells. LAMIN A/C was used as a loading control. **c** Total numbers of peptides detected by mass spectrometry (2nd and 3rd columns from the left) and normalized to the number of the corresponding RECQ4 peptides (4th and 5th columns) for each of the indicated proteins co-purified with FLAG-RECQ4 from the CB fractions of RECQ4 WT or Q757X mutant expressing cells. Complete mass spectrometry list is included as Supplementary Data 1. *n* = 1 (biologically independent samples). **d** Representative western blot analysis of the indicated proteins co-purified with FLAG-RECQ4 from the CE (left) and CB (right) fractions of RECQ4 WT or Q757X mutant expressing cells. Source data are provided as a Source data file.

We next determined the mechanism by which the RECQ4 C-terminus limits origin firing by analyzing the chromatin-bound (CB) RECQ4 replication complexes formed in RECQ4 KD cells expressing FLAG-RECQ4 WT or Q757X mutant proteins. When we fractionated whole-cell extracts (WCEs) into cytoplasmic (CE), nucleoplasmic, and CB fractions, we observed a modest increase (25-30%) in the amount of Q757X mutant protein in the CB fraction compared to WT protein (Fig. 2a). Replication complex assembly to support DNA synthesis is a multi-step process initiated by the formation of a pre-replication complex (pre-RC) at the replication origin^28^. We found that components of the pre-RC (e.g., CDC6 and CDT1) and several components of the replication complex (e.g., MCM2-7, GINS, and DNA polymerase δ) were enriched by more than 2-fold in the CB fraction of Q757X mutant cells compared to WT cells (Fig. 2b). We also immunopurified FLAG-RECQ4 WT and Q757X mutant proteins from the CB fractions using M2 agarose beads conjugated with anti-FLAG antibody and identified their associated proteins by mass spectrometry (Supplementary Data 1). After normalizing to the FLAG-tagged bait proteins, we found that replication factors co-purified with FLAG-RECQ4 Q757X increased by as much as 15-fold compared to those co-purified with FLAG-RECQ4 WT protein, as confirmed by both mass spectrometry (Fig. 2c; Supplementary Data 1) and western blot analysis (Fig. 2d). These increases were specific to the CB fraction, as demonstrated by comparison of the replication factors MCM2-7 (e.g., MCM2 and MCM7), CDC45, and GINS (e.g., GINS2 and GINS4) in FLAG-RECQ4 complexes purified from the CE (left) and the CB (right) fractions (Fig. 2d). These results indicate that the RECQ4 C-terminus limits formation of the pre-RC and subsequently the replisome on chromatin.

In addition to DNA replication factors^10^, we previously detected components of the APC/C E3 ubiquitin ligase in mass spectrometry analyses of FLAG-RECQ4 complexes purified from human cell extracts (Supplementary Data 2)^10,17^, but the physiological significance of this association was not immediately clear. In the current study, we compared FLAG-RECQ4 WT and Q757X mutant complexes on chromatin using mass spectrometry (Fig. 2c; Supplementary Data 1) and western blot analysis (Fig. 2d) and observed that interactions with APC/C subunits were enhanced by as much as 35-fold in the Q757X mutant complex. In contrast, interactions with ATP citrate lyase (ACLY), ubiquitin-activating enzyme E1 homolog A (UBA1), and mitotic checkpoint protein BUB3, all of which also co-purified with RECQ4, were significantly weakened by the Q757X mutation (Fig. 2c, d). Furthermore, increased binding of APC/C to the RECQ4 Q757X mutant was specific to the CB fraction and was not present in the CE fraction, as validated by western blot analysis using antibodies specific to the APC1 and APC5 subunits (Fig. 2d). On the other hand, only the RECQ4 WT interaction with BUB3, which has a role in regulating APC/C^29,30^, took place in the CB fraction; RECQ4 WT interactions with UBA1 and ACLY were detected in the CE fraction (Fig. 2c, d).

**Fig. 3:**
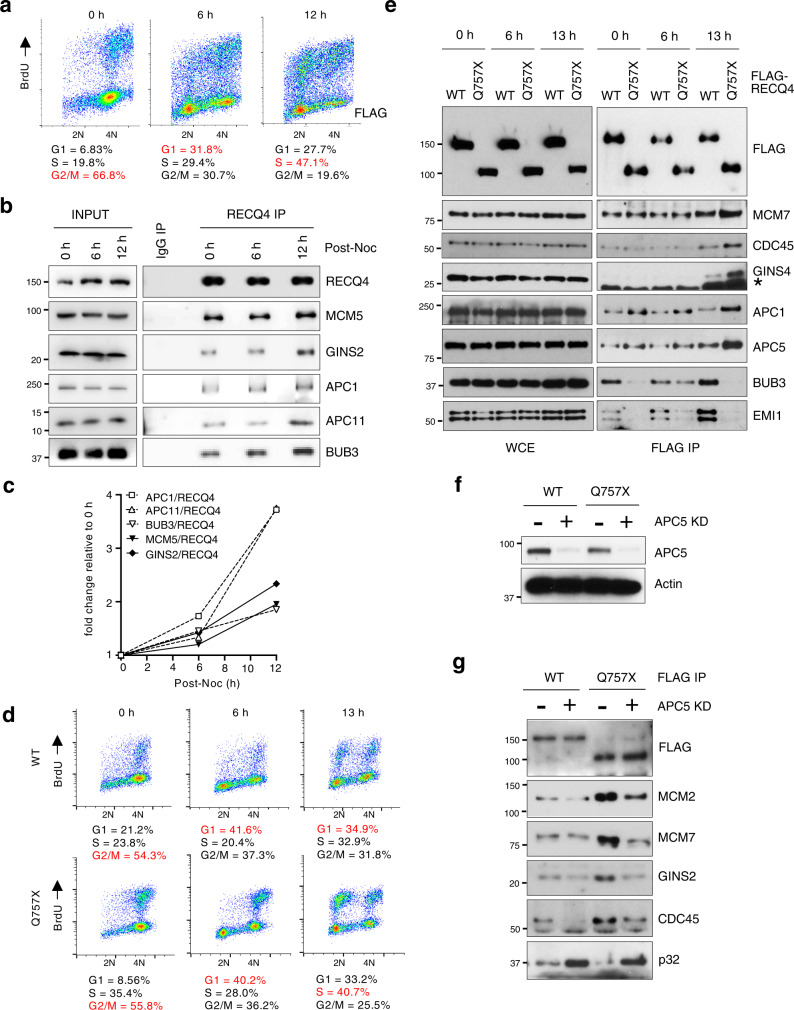
APC/C facilitates RECQ4 Q757X replication complex formation. **a** Flow cytometry analysis of parental HEK293 cells after release from nocodazole blocking at the indicated time points. The cells were labeled with BrdU and co-stained for BrdU incorporation (Y-axis) and DNA content (propidium iodide [PI], X-axis). Percentages of cells in G_1_, S, and G_2_/M phases are shown. Percentages of cells in G_1_, S, and G_2_/M phases are shown, with the highest percentage in each time point indicated in red. **b** Representative western blot analysis of the indicated proteins in whole-cell extracts (WCE; input; left) prepared from cells shown in (**a**) and of proteins co-immunoprecipitated (right) with RECQ4 using a rabbit anti-RECQ4 antibody at the indicated time points post-nocodazole (Noc) release. **c** Quantification of fold change in the amount of the indicated co-purified proteins normalized to the bait protein RECQ4 compared to 0 h in (**b**). A higher value on the Y-axis corresponds to an increasing interaction. **d** Flow cytometry analysis of RECQ4 knockdown (KD) HEK293 cells stably expressing FLAG-tagged RECQ4 wild type (WT, top) or Q757X mutant (bottom) protein after release from nocodazole blocking at the indicated time points. Percentages of cells in G_1_, S, and G_2_/M phases are shown, with the highest percentage in each time point indicated in red. **e** Representative western blot analysis of the indicated proteins in WCE (left) prepared from cells shown in (**d**) and of proteins co-purified with FLAG-RECQ4 WT or Q757X mutant proteins using M2 agarose beads (right) at the indicated time points post-nocodazole release. * indicates non-specific bands cross-reacting with the GINS4 antibody. **f** Representative western blot analysis of APC5 in FLAG-RECQ4 WT or Q757X mutant expressing cells treated with or without shRNA specifically targeting APC5 [APC5 knockdown (KD)]. Actin was used as a loading control. **g** Representative western blot analysis of the indicated proteins co-purified with FLAG-RECQ4 WT or Q757X mutant proteins prepared from cells shown in (**f**). Source data are provided as a Source data file.

Because APC/C is an important E3 ubiquitin ligase that controls both G_1_/S transition and mitosis^31^, we next wished to confirm that endogenous RECQ4 interacts with APC/C and determine whether this interaction is cell-cycle regulated. To test this, we synchronized parental HEK293 cells with nocodazole; after release from nocodazole block, we purified endogenous RECQ4 complexes from WCE using an antibody specific to RECQ4 (Fig. 3a). We found that APC/C co-immunoprecipitated with endogenous RECQ4, and that similar to its interaction with replication factors (e.g., MCM5 and GINS2)^10^, the interaction of RECQ4 with APC/C (e.g., APC1 and APC11) peaked at 12 h post-nocodazole release when cells were transitioning from G_1_ to S phase (Fig. 3b, right panels, 3c). Although BUB3 functions as an APC/C inhibitor during M phase^30,32^, our cell cycle analysis showed that the interaction with BUB3 also increased as more cells entered S phase (Fig. 3b, c). When we repeated the experiment in RECQ4 KD HEK293 cells stably expressing FLAG-RECQ4 WT, we observed similar kinetics of APC/C and BUB3 interactions during the cell cycle (Fig. 3d, e), despite exogenous RECQ4 being constitutively expressed throughout the cell cycle (Fig. 3d, e, left panel), unlike endogenous RECQ4 (Fig. 3b, input). Importantly, we observed a maximum increase in the binding of RECQ4 Q757X mutant vs. WT protein to replication factors and APC/C during S phase (i.e., 13 h; Fig. 3e). When we assessed EMI1, an APC/C inhibitor that acts during S phase to prevent re-replication^33–35^, we found that EMI1 also co-purified with RECQ4 WT mainly during S-phase (Fig. 3e). For both BUB3 and EMI1, the interaction was impaired by the Q757X mutation (Fig. 3e).

Because APC/C and various replication factors shared similar kinetics in their interactions with RECQ4, we next asked if APC/C is responsible for enhanced interaction of the Q757X mutant with replication factors. For this, we depleted the APC5 subunit of the APC/C complex by expressing an shRNA specific to APC5 in both the RECQ4 WT and Q757X mutant cells (Fig. 3f). We found that depleting APC5 indeed reversed the enhanced interaction of RECQ4 Q757X mutant with replication factors (MCM2, MCM7, GINS, CDC45) to a level comparable to that of the WT protein (Fig. 3g). These results together indicate that APC/C interacts with RECQ4 during S phase to enhance replisome assembly, and this function is self-limited by the RECQ4 C-terminus.

### Multiple domains within the RECQ4 N-terminus interact with APC/C

**Fig. 4:**
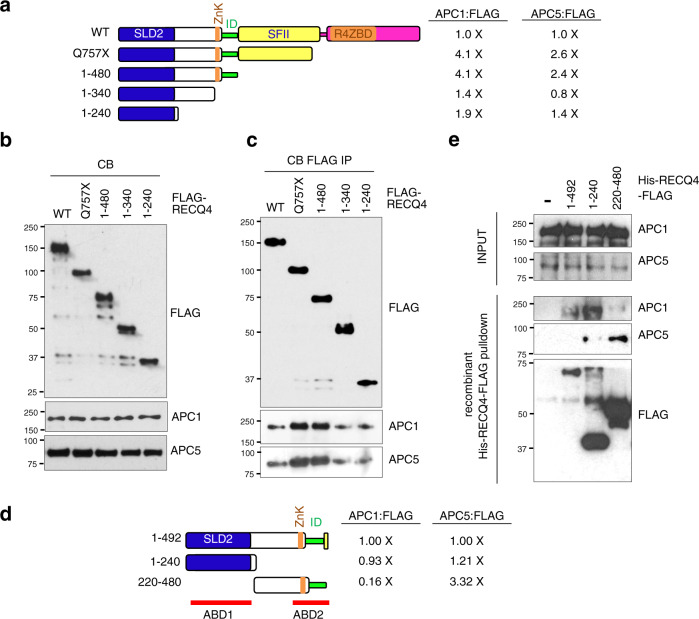
Multiple domains within the RECQ4 N-terminus interact with APC/C. **a** (left) A schematic diagram of RECQ4 wild type (WT), Q757X, and indicated N-terminal fragments. (right) Fold changes in the amount of APC1 or APC5 pulled down, normalized to the bait FLAG-RECQ4 proteins relative to FLAG-RECQ4 WT based on the western blot analysis shown in (**c**). **b** Representative western blot analysis of the indicated proteins in chromatin-bound (CB) fractions prepared from RECQ4 knockdown (KD) HEK293 cells transiently overexpressing the indicated FLAG-RECQ4 constructs. APC1 and APC5 were used as loading controls. **c** Representative western blot analysis of APC1 and APC5 co-purified with the indicated FLAG-RECQ4 constructs using the CB fractions shown in (**b**). **d** (left) A schematic diagram of the His-RECQ4-FLAG constructs for expression and purification from *E. coli*. (right) Fold changes in the amount of APC1 or APC5 pulled down from the CB fraction prepared from parental HEK293 cells normalized to the recombinant RECQ4 bait protein fragments relative to the RECQ4_1-492_ fragment based on the western blot analysis shown in (**e**). APC/C-binding-domain 1 (ABD1) and ABD2 are labeled with red lines. **e** Representative western blot analysis to detect APC1 and APC5 from the CB fraction prepared from parental HEK293 cells (input; top panels) and those bound to M2 agarose in the presence of the indicated recombinant His-RECQ4-FLAG proteins (lower 3 panels). Source data are provided as a Source data file.

We next mapped the residues present in the first 757 amino acids of RECQ4 that contribute to its association with APC/C. To do this, we transiently overexpressed various FLAG-tagged RECQ4 deletion mutants to achieve similar expression levels in RECQ4 KD HEK293 cells (Fig. 4a) and confirmed that all RECQ4 fragments were detected in the CB fraction (Fig. 4b). When normalized to FLAG-RECQ4 proteins immunopurified on M2 agarose beads, we found that an N-terminal RECQ4_1-480_ fragment without the conserved SFII domain showed a similar increase (4.1-fold for APC1 and 2.4-fold for APC5) as the Q757X mutant (4.1-fold for APC1 and 2.6-fold for APC5) in binding to components of APC/C compared to the WT RECQ4 (Fig. 4a, c). However, removing the residues between 340 and 480 (i.e., FLAG-RECQ4_1-340_) reduced the interaction of RECQ4 with APC/C by approximately 3-fold compared to FLAG-RECQ4_1-480_ (Fig. 4c), even though these two fragments were present at similar levels in the CB fractions (Fig. 4b). A RECQ4_1-240_ fragment that contains only the SLD2 domain showed similar APC/C binding efficiency as the RECQ4_1-340_ fragment. This result suggests that the RECQ4 N-terminus contains two APC/C-binding domains (ABD), with ABD1 located within SLD2, and ABD2 overlapping with residues 340–480 (Fig. 4d). Interestingly, ABD2 contains the 44 residues missing in the cancer-prone ID mutation (Fig. 4d). Previously, we showed that the ID motif is required for RECQ4 interaction with p32^17^, which suggests that the p32 interacting site may overlap with that of APC/C. Indeed, we found that depleting APC/C in RECQ4 KD HEK293 cells expressing FLAG-RECQ4 WT or Q757X mutant increased the amount of p32 co-purified with both WT and Q757X mutant proteins (Fig. 3g, bottom panel).

We next wished to confirm that ABD1 and ABD2 are both involved in APC/C binding and to exclude the possibility that differential binding to APC/C by distinct FLAG-RECQ4 fragments transiently overexpressed in RECQ4 KD HEK293 cells was due to changes in cell cycle or protein localization. For this, we performed in vitro pull-down experiments using recombinant His-tagged, FLAG-tagged RECQ4 N-terminal fragments (Fig. 4d) purified from *E. coli* and conjugated to M2 agarose beads to pull down APC/C from the CB fraction prepared from parental HEK293 cells. After normalizing to the His-RECQ4-FLAG bait protein on the beads, we confirmed that both RECQ4_1-240_, which contains ABD1, and RECQ4_220-480_, which contains ABD2, pulled down components of APC/C, but with different binding preferences. Specifically, the fragment containing ABD1 preferentially pulled down APC1, whereas that containing ABD2 preferentially pulled down APC5 (Fig. 4e). Interestingly, recombinant RECQ4_220-480_ pulled down 3-fold more APC5 than RECQ4_1-492_ (Fig. 4e). It is possible that the presence of ABD1 affects ABD2 access to APC5 but may be altered by additional regulations, such as post-translational modifications that are not present in the recombinant proteins generated in *E. coli*. In summary, these results indicate that the RECQ4 N-terminus contains two ABDs that bind to different APC/C subunits.

### RECQ4 ABD2-APC/C interaction promotes replication complex assembly

**Fig. 5:**
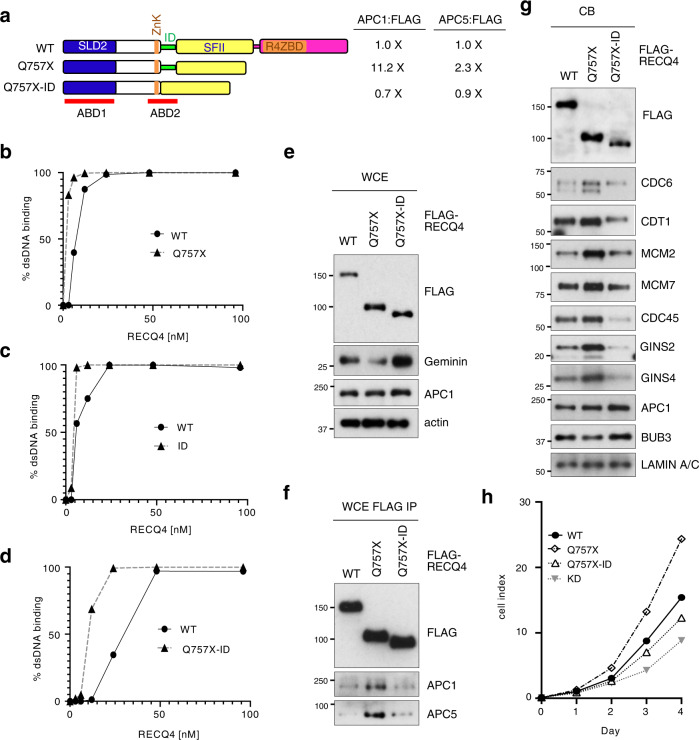
RECQ4 N-terminal interaction with APC/C promotes replisome assembly. **a** (left) A schematic diagram of RECQ4 wild type (WT), Q757X, and Q757X-internal-deletion (ID) double mutant. (right) Fold changes in the indicated co-purified proteins normalized to the bait FLAG-RECQ4 proteins relative compared to the FLAG-RECQ4 WT immunoprecipitation (IP) based on the western blot analysis shown in (**f**). **b**–**d** Quantification of DNA binding efficiency of recombinant His-tagged, FLAG-tagged RECQ4 Q757X (**b**), ID (**c**), or Q757X-ID (**d**) compared to WT RECQ4. **e** Representative western blot analysis of the indicated proteins in whole-cell extracts (WCE) prepared from RECQ4 knockdown (KD) HEK293 cells stably expressing RECQ4 WT, Q757X, or Q757X-ID. Actin was used as a loading control. **f** Representative western blot analysis of the indicated proteins co-immunopurified with FLAG-RECQ4 WT, Q757X, or Q757X-ID proteins prepared from WCE shown in (**e**). **g** Representative western blot analysis of the indicated proteins in chromatin-bound (CB) fractions prepared from RECQ4 WT, Q757X, or Q757X-ID expressing cells. LAMIN A/C was used as a loading control. **h** Representative real-time cell growth assays to measure cell growth rate of RECQ4 KD cells with or without stable expression of FLAG-RECQ4 WT, Q757X, or Q757X-ID mutant. Source data are provided as a Source data file. *n* = 3 (biologically independent samples). Data are presented as mean values ± SD.

Our domain analysis showed that the RECQ4 C-terminus negatively regulates ABD2 interaction with APC/C. Therefore, we next tested if disrupting the interaction of RECQ4 ABD2 with APC/C is sufficient to reverse the deregulation of replication complex formation by the Q757X mutation. ABD2 contains a zinc knuckle (ZnK; Fig. 5a) that is involved in nucleic acid binding^36^. To avoid disrupting ZnK, we generated a RECQ4 Q757X-ID double mutant, in which only the residues corresponding to the ID motif and downstream of the ZnK were removed from the Q757X fragment (Fig. 5a). Using purified recombinant His-RECQ4 WT, Q757X, ID, and Q757X-ID mutant proteins from *E. coli* (Supplementary Figs. 3a, 3b), we confirmed that deleting ID in either the WT or Q757X mutant construct did not interfere with DNA binding (Fig. 5b–d; Supplementary Fig. 3c–e). We next stably expressed the Q757X-ID construct in RECQ4 KD HEK293 cells and compared it to those stably expressing the RECQ4 WT and Q757X proteins (Fig. 5e, top panel). We found that introducing the additional ID mutation reduced the ability of the Q757X mutant protein to interact with APC/C (e.g., APC1 and APC5) by as much as 16.5-fold (Fig. 5f), even though there was only a 30% decrease in the amount of Q757X-ID mutant binding to chromatin (Fig. 5g, top panel). In addition, the levels of pre-RC factors (e.g., CDC6 and CDT1) and components of the replication complex (e.g., MCM2, MCM7, CDC45, GINS2, and GINS4) on chromatin were reduced in Q757X-ID double mutant cells by more than 2-fold compared to those expressing the Q757X single mutant (Fig. 5g). Consistent with this, we also observed that Geminin was reduced in Q757X mutant cells, but the reduction was reversed by the Q757X-ID double mutation (Fig. 5e). Hence, Q757X-ID double mutant cells lost the growth advantage found in Q757X mutant cells over WT cells (Fig. 5h).

### RECQ4-APC/C interaction facilitates the degradation of Geminin

**Fig. 6:**
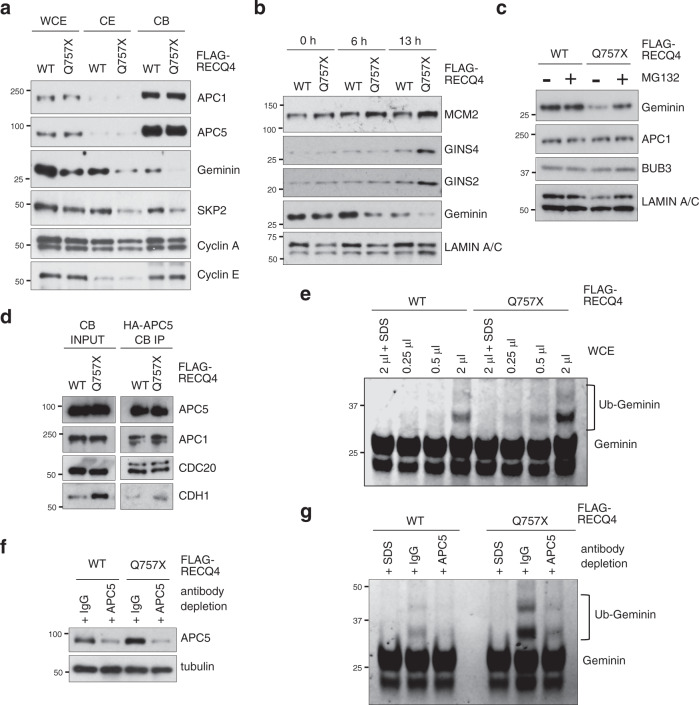
RECQ4 enhances APC/C E3 ubiquitin ligase activity. **a** Representative western blot analysis of the indicated proteins in whole-cell extracts (WCE), cytoplasmic (CE), and chromatin-bound (CB) fractions prepared from RECQ4 knockdown (KD) HEK293 cells stably expressing FLAG-tagged RECQ4 wild type (WT) or Q757X mutant protein. **b** Representative western blot analysis of the indicated proteins in CB fractions prepared from RECQ4 WT or Q757X mutant expressing cells at the indicated time points post-nocodazole release. LAMIN A/C was used as a loading control. **c** Representative western blot analysis of the indicated proteins in CB fractions prepared from RECQ4 WT or Q757X mutant expressing cells with or without MG132 treatment to inhibit proteasome activity. LAMIN A/C was used as a loading control. **d** Representative western blot analysis of the indicated proteins in CB fractions (left) and in immunopurified APC5 protein complexes from the CB fractions (right) prepared from RECQ4 WT or Q757X mutant expressing cells transfected with HA-tagged APC5. **e** Representative in vitro ubiquitination assays using purified recombinant FITC-labeled Geminin in the presence of indicated amounts of WCEs prepared from equal numbers of RECQ4 WT or Q757X mutant expressing cells. SDS was added as a control to denature all proteins. Geminin and its ubiquitinated state were visualized by Odyssey Fc (LI-COR) at 600 nm. **f** Representative western blot analysis of APC5 in the WCE prepared from RECQ4 WT or Q757X mutant expressing cells with control or APC5 depletion using IgG or an antibody specific to APC5. Tubulin was used as a loading control. **g** Representative in vitro ubiquitination assays using purified recombinant FITC-labeled Geminin in the presence of equal amounts of WCEs with or without APC5 depletion shown in (**f**). Source data are provided as a Source data file.

During G_1_ phase and G_1_/S transition, APC/C is activated by the adaptor protein CDH1 to promote pre-RC assembly and origin firing by degrading the replication inhibitor Geminin^34,37^. Because we found that the amount of Geminin (Fig. 5e) was inversely proportional to the interaction of APC/C with RECQ4 (Fig. 5f), we asked if RECQ4-APC/C interaction facilitates Geminin degradation. We first fractionated RECQ4 KD HEK293 cells stably expressing FLAG-RECQ4 WT or Q757X mutant and confirmed that Geminin was proportionally reduced in the CB fraction prepared from Q757X mutant cells compared to WT cells (Fig. 6a). The reduction was most noticeable at 6 h (2-fold) and 13 h (3-fold) post-nocodazole release, when the cells were in G_1_ and S phase, respectively (Fig. 6b). We further determined that Geminin was stabilized in Q757X mutant cells by treating the cells with the proteasome inhibitor MG132; the amount of Geminin was restored to a similar level as that in WT cells (Fig. 6c), confirming that expression of reduced Geminin in the Q757X mutant cells was due to proteasome-mediated protein degradation.

The increased degradation of Geminin in Q757X mutant cells led us to test whether APC/C co-activator expression (e.g., CDH1 and CDC20) is increased in Q757X mutant cells. We detected increased CDH1 by 3.5-fold in the CB fraction of Q757X mutant cells compared to WT cells (Fig. 6d). When we used an antibody specific to the HA-tag to immunopurify the APC/C complex from RECQ4 WT and Q757X mutant cells expressing an HA-tagged APC5 subunit, we found that CDH1 association with APC/C was enhanced (2-fold) by the RECQ4 Q757X mutation (Fig. 6d). On the other hand, Q757X mutation had little effect on the level of CDC20 (Fig. 6d), which primarily functions to activate APC/C during mitosis^38^. RECQ4 C-terminal deletion also did not enhance CDC20 association with APC/C in the CB fraction (Fig. 6d). An increase in APC/C-CDH1, but not APC/C-CDC20, complexes is consistent with the fact that expression of S-phase kinase-associated protein 2 (SKP2), which is an APC/C-CDH1 substrate^39^, was also lower in the Q757X mutant cells compared to WT cells (Fig. 6a). In contrast, cyclin A degradation, which is mediated primarily by APC/C-CDC20^32^, was not affected by the RECQ4 Q757X mutation (Fig. 6a). Although CDC6 is also a known APC/C substrate, its stability was not reduced by the Q757X mutation (Fig. 2b). This may be because CDC6 is protected from APC/C-CDH1-mediated degradation by CDK-dependent phosphorylation in cycling cells^40^.

To determine if APC/C E3 ubiquitin ligase activity is affected in Q757X mutant cells, we performed in vitro ubiquitination assays using purified recombinant Geminin to measure APC/C activity in WCEs prepared from RECQ4 WT and Q757X mutant cells. When we incubated purified recombinant Geminin proteins with equal amounts of RECQ4 WT or Q757X mutant WCE, we observed more ubiquitinated Geminin proteins in the reactions containing Q757X mutant WCE than those with WT WCE (Fig. 6e). We further depleted APC/C from both WCEs using an anti-APC5 antibody (Fig. 6f) and confirmed that APC/C-depleted WCEs from both RECQ4 WT and Q757X mutant cells exhibited reduced ubiquitination activity on the recombinant Geminin substrate (Fig. 6g).

During S phase, Geminin expression is restored by inactivation of APC/C to prevent re-replication during the mitotic cycle^33^. In addition, CDT1 is targeted for degradation by SKP2 to prevent re-replication^41–43^. Geminin expression in Q757X mutant cells continued to remain lower than that in WT cells during S phase (Fig. 6b), raising the possibility that Q757X mutation increases the risk of re-replication. Developmental signaling or external stress, such as G_2_ arrest by nocodazole, is known to induce re-replication by activating APC/C^44,45^. Consistent with this, by 15 h post-nocodazole release, we observed that not only were more Q757X mutant cells replicating DNA (i.e., greater % in S phase) than were WT cells, but that the BrdU-positive cell population containing more than 4N DNA was increased in Q757X vs. WT cells, likely due to additional rounds of DNA synthesis without cell division (Supplementary Fig. 4). In summary, these results demonstrate that the RECQ4 C-terminus restricts the RECQ4 N-terminus from binding to APC/C, thereby limiting APC/C-mediated degradation of Geminin and pre-RC chromatin association to prevent DNA replication initiation and re-replication.

### EMI1 interaction with RECQ4 C-terminus is inversely correlated with RECQ4-APC/C interaction

**Fig. 7:**
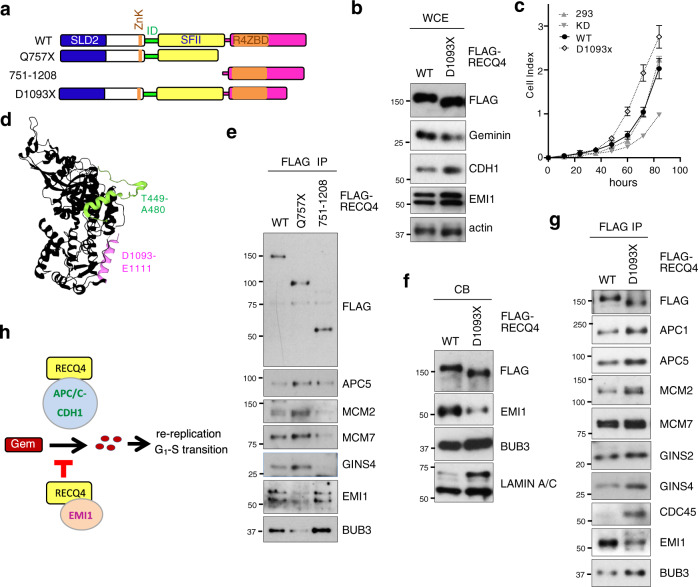
RECQ4 C-terminal interaction with EMI1 is inversely correlated with RECQ4 N-terminal interaction with APC/C. **a** A schematic diagram of RECQ4 wild type (WT), Q757X mutant, C-terminal fragment (751–1028), and D1093X mutant. **b** Representative western blot analysis of the indicated proteins in whole-cell extracts (WCE) prepared from RECQ4 WT and D1093X expressing cells. Actin was used as a loading control. **c** Representative real-time cell growth assays to measure cell growth rate of HEK293 or RECQ4 knockdown (KD) cells with or without stable expression of FLAG-RECQ4 WT or D1093X mutant. *n* = 4 (biologically independent samples), except HEK293 control cells, where *n* = 3 (biological independent samples). Data are presented as mean values ± SD. **d** Structure of RECQ4_427-1116_ (5LST) drawn using RProtein Data Bank. The residues visible in the structure between T449 and A480 that are part of ABD2 and important for APC/C interaction are shown in green. Visible residues (D1093 to E1111) missing in the D1093X mutation involved in EMI1 interaction are shown in pink. **e** Representative western blot analysis of the indicated proteins co-purified with FLAG-RECQ4 WT, Q757X, or 751–1208 from RECQ4 KD HEK293 cells stably expressing RECQ4 WT or truncation mutants shown in (**a**). **f** Representative western blot analysis of the indicated proteins in chromatin-bound (CB) fractions prepared from RECQ4 WT and D1093X expressing cells. LAMIN A/C was used as a loading control. **g** Representative western blot analysis of the indicated proteins co-purified with FLAG-RECQ4 WT or D1093X proteins from CB fractions prepared from the cells shown in (**f**). Source data are provided as a Source data file. **h** Schematic diagram of the proposed role of RECQ4-APC/C interaction in replication initiation. See “Discussion” for details.

We next explored the mechanism by which the RECQ4 C-terminus negatively regulates the interaction between the RECQ4 N-terminus and APC/C. Similar to the Q757X mutation, the D1093X clinical mutation, which deletes the terminal 115 amino acids of RECQ4 (Fig. 7a), has been linked to RTS and osteosarcoma^46^. When we stably expressed a FLAG-tagged RECQ4 D1093X mutant protein at a similar level as WT RECQ4 in RECQ4 KD HEK293 cells (Fig. 7b), we found that D1093X mutant cells also exhibited a faster cell growth rate than WT cells (Fig. 7c). Importantly, D1093X mutant expression led to reduced expression of Geminin, accompanied by increased expression of CDH1, relative to that in WT cells (Fig. 7b). Interestingly, the crystal structure of human RECQ4_427-1116_ fragment^47^ indicates that residues containing the ID motif that are involved in APC/C binding and visible in the structure (Fig. 7d, T449-A480 shown in green) are in close proximity and facing the same surface as the C-terminal residues between D1093 and E1111 (Fig. 7d, α-helix shown in pink) that are missing in the D1093X mutation. Therefore, we tested whether a protein–protein interaction with the terminal 115 residues of RECQ4 blocks the nearby ID motif and limits its accessibility to APC/C, thereby weakening RECQ4-APC/C interaction. Because Q757X mutation impaired RECQ4 binding to both APC/C inhibitors EMI1 and BUB3 (Fig. 3e), we next determined if EMI1 or BUB3 interacts with the terminal 115 residues of RECQ4 to block the ID interaction with APC/C and replication complex assembly. We first expressed a RECQ4 C-terminal fragment containing residues 751 to 1208 (Fig. 7a) and confirmed that both EMI1 and BUB3, but not replication factors (e.g., MCM2, MCM7, GINS4), interacted primarily with the C-terminus of RECQ4 (Fig. 7e). Although the level of D1093X mutant on chromatin did not increase compared to WT (Fig. 7f), components of APC/C (e.g., APC1 and APC5) and replication factors (e.g., MCM2, MCM7, GINS2, GINS4, and CDC45) were enriched in FLAG-RECQ4 D1093X complexes immunopurified from the CB fraction (Fig. 7g), indicating that the last 115 residues of RECQ4 are involved in suppressing APC/C interaction with the ID motif in the N-terminus. Importantly, only the EMI1 interaction, and not the BUB3 interaction, was weakened by the D1093X mutation and inversely correlated with the RECQ4-APC/C interaction (Fig. 7g). These results support a model in which the EMI1-interacting residues on RECQ4 are spatially in proximity to those important for APC/C interaction, and RECQ4-EMI1 interaction may impose a steric hindrance to prevent RECQ4 interaction with the APC/C complex (Fig. 7d). Our analysis of the D1093X mutant also suggests that while there is an increase in Q757X mutant proteins on chromatin compared to WT RECQ4 proteins, this increase is not required for enhancing RECQ4-APC/C interaction.

## Discussion

We identified a chromatin-specific interaction that occurs between RECQ4 and APC/C through the N-terminus of RECQ4 and showed that this interaction promotes replication complex assembly and replication initiation. We showed that maximum binding of RECQ4 to APC/C on chromatin requires both ABD1 overlapping with the SLD2 and ABD2 containing the ID motif within the RECQ4 N-terminus. Because APC/C is a megadalton, multi-protein complex, it is unlikely that ABD1 and ABD2 independently bind to two separate APC/C complexes. As these two domains showed binding preferences for distinct APC/C subunits in vitro, we propose that maximum binding is achieved by the interaction of ABD1 and ABD2 with different components of the same APC/C complex to stabilize the interaction. Given that ABD1 contains SLD2 that interacts with replication factors^10^, it is also possible that the ABD1-APC/C association is indirect via their mutual interactions with replication factors. We previously showed that RECQ4 ID mutant proteins not only accumulate in the mitochondria due to impaired interaction with p32 but also exhibit reduced nuclear localization^17^. Given that proteins can shuttle between the nucleus and mitochondria^48^, our previous study suggested that the reduction in nuclear RECQ4 ID mutant proteins is a consequence of imbalanced shuttling. Our current study provides an alternative but not mutually exclusive possibility that the reduction in nuclear RECQ4 ID mutant proteins may also be due to a reduced affinity for APC/C.

Our study further identified an inhibitory activity within the RECQ4 C-terminal 115 residues (1093–1208) that blocks the APC/C interaction with RECQ4 ABD2. Because these C-terminal residues are spatially located in proximity to ABD2, we suggest that a protein–protein interaction with these C-terminal residues blocks the nearby RECQ4 ABD2. Both BUB3 and EMI1 function as APC/C inhibitors^49,50^. However, only interaction with EMI1 was mapped to the terminal 115 residues of RECQ4 and inversely correlated with the interaction of RECQ4 with APC/C. EMI1 may interact with the RECQ4 C-terminus to block APC/C from accessing ABD2; these mutually exclusive interactions suggest a possible new mechanism by which EMI1 inhibits APC/C activity during S phase. In cells expressing a RECQ4 C-terminal truncation mutant, such as Q757X or D1093X, RECQ4-EMI1 interaction was weakened, allowing the RECQ4 N-terminus to increase interaction with and activate APC/C, thereby enhancing Geminin degradation and DNA replication initiation during S phase.

Negative regulation of DNA replication initiation by the RECQ4 C-terminus may act to prevent unscheduled origin firing during G_1_/S transition (Fig. 7h). The increased origin firing frequency we observed in Q757X mutant cells is consistent with the accelerated cell cycle progression of both G_1_ and S phases, resulting in the faster growth rate observed in RECQ4 Q757X mutant vs. WT cells. In addition, APC/C-mediated re-replication can be induced during development and tissue regeneration in response to developmental signaling or external stress^44,45^. Re-replication has emerged as a key step during differentiation of multiple tissues^51^, and *Drosophila* RECQ4 has been shown to promote re-replication during development^52^. Because we observed an increased number of RECQ4 Q757X mutant vs. WT cells with DNA index >4N, likely due to additional rounds of DNA replication without cell division, this suggests that negative regulation of replication initiation by the RECQ4 C-terminus is related to suppressing re-replication (Fig. 7h). Our study also provides an explanation of why mouse embryonic fibroblasts isolated from mice expressing only the N-terminal RECQ4 domain were hyperploid^6^. Geminin and SKP2 prevent re-replication by limiting replication to one round of origin firing per cell cycle^41–43,53^. It would be of great interest for future studies to determine if RECQ4 facilitates re-replication via the activation of APC/C to degrade Geminin and SKP2 during tissue differentiation and promote tissue development. If so, it would be important to determine how cells switch between RECQ4-APC/C and RECQ4-EMI1 complexes during these processes, and if this regulation involves post-translational modifications. It is worth noting that APC2, a component of the APC/C complex, has been detected in a synthetic genetic array analysis of the yeast Hrq1 (RECQ4 homolog) protein complex^54^. It remains to be determined if the role of RECQ4-APC/C interaction in replication initiation is conserved in yeast.

Clinical analyses indicate that more than 50% of RTS cases are consequences of mutations in the *RECQ4* gene^55,56^. Recently, *ANAPC1*, which encodes APC1, the largest subunit of APC/C, was also identified as a genetic risk factor that when mutated, contributes to RTS pathogenesis in the remaining RTS patient population with intact *RECQ4* gene copies^20^. Our study used an unbiased proteomic approach to uncover mechanistic insights into the functional link between these two RTS factors in regulating DNA replication initiation. The significance of this link is underscored by the fact that RECQ4 clinical mutations alter RECQ4 interaction with APC/C. It would be of great interest to determine if RECQ4 clinical mutations induce deregulation of the RECQ4-APC/C interaction, leading to abnormal replication origin firing and high DNA content, which contributes to developmental abnormalities associated with RTS and cancer pathogenesis.

Our mass spectrometry analysis also identified changes in RECQ4 cytoplasmic interactions by the Q757X mutation. While beyond the scope of the current study, it would be of great interest to explore the functional significance of these interactions. For example, UBA1 is a ubiquitin E1 ligase, and its interaction with RECQ4 may regulate ubiquitination and protein degradation in other cellular processes. In addition, RECQ4 interacts with ACLY, a key enzyme to support Warburg effect^57^. We previously showed that RECQ4 ID mutant proteins accelerated cell growth by inducing Warburg effect through mitochondrial dysfunction^17^. Further work is needed to determine the contribution of RECQ4-ACLY interaction to Warburg effect. Because this interaction is impaired by the Q757X mutation in cytoplasm, it is possible that Q757X-ID double mutant does not share the same growth advantage as either Q757X or ID mutant over WT RECQ4, because it fails to enhance origin firing as well as Warburg effect.

## Methods

### Plasmids

A pCMV-FLAG-RECQ4 plasmid was previously constructed^10^. pMCV-FLAG-RECQ4-Q757X and pCMV-FLAG-RECQ4-D1093X plasmids were generated by site-directed mutagenesis of the pCMV-FLAG-RECQ4 plasmid. A pCMV-RECQ4-Q757X-ID plasmid was generated by the combination of pCMV-FLAG-RECQ4-ID^17^ and pCMV-FLAG-RECQ4-Q757X at the BamHI site. pCMV-FLAG-RECQ4(1–480), pCMV-FLAG-RECQ4(1–340), pCMV-FLAG-RECQ4(1–240), and FLAG-RECQ4(751–1208) plasmids were prepared by PCR amplification of cDNAs and cloning into the NdeI and XhoI sites of a pCMV-FLAG vector. To generate pCMV-HA-APC5 plasmid, APC5 cDNA was PCR-amplified from a Hela cDNA library and cloned into the XhoI and EcoRI sites of a pCMV-HA vector, which was modified from the pCMV-FLAG vector. A Tet-pLKO-shAPC5 plasmid was constructed by cloning annealed shRNA sequences into a Tet-pLKO-Puro vector (Addgene, #21915). Ubiquitin (Ubi) and Geminin (GMNN) cDNA were PCR-amplified from a Hela cDNA library. A pET-16b-Ubi plasmid was constructed by inserting ubiquitin cDNA into a pET-16b vector (Novagen) with NdeI and XhoI sites. A pTXB1-GMNN plasmid was constructed by inserting Geminin cDNA into a pTXB1 vector (NEB) with NdeI and XhoI sites. Primer sequences were as follows: RECQ4_fwd 5’-GGTCGTCATATGGAGCGGCTGCGGGACGTGCGGGAG-3′; RECQ4_rv 5′-CCG CTCGAGAGCGGGCCACCTGCAGGAG-3′; R751_fwd 5′-GGATCATATGCGGG AACGGCGGGTACAGC-3′; 1-340_rv 5′-CC GCTCGAGGTGCAGGGGGGCTGTG CCT-3′; 1-480_rv 5′-CCGCTCGAGGGCTTG GTGCCCCAGCTGCTC-3′; Q757X 5′-GGGAACGGCGGCGGGTATAGCGAGCCTTCATGCAG-3′; D1093M 5′-CCC TGCCTGGAGCAGCAGATGAGGAGCGCAGCAC-3′; APC5_fwd 5′-CCGCTCGA GATGGCCAGCGTCCACGAGAGCCT-3′; APC5_rv 5′-CGGAATTCCTAGAGAT GGTTTATCAAGGGTACC-3′; shAPC5_fwd 5′-CCGGTCTCTCCCTATAAATGAT GTACTCGAGTACATCATTTATAGGGAGA GATTTTTG-3′; shAP5_rv 5′-AATTC AAAAATCTCTCCCTATAAATGATGTACTCGAGTACATCATTTATAGGGAGAGA-3′; Ubi_fwd 5′-GGAATTCCATATGCAGA TCTTCGTGAAAACC-3′; Ubi_rv 5′-CCGCTCGAGCTAACCACCTCTCAGACGCAG-3′; GMNN_fwd 5′-GGAATTCC ATATGAATCCCAGTATGAAGCAGAAAC-3′; GMNN_rv 5′-CCGCTCGAGAGC TATACATGGCTTTGCATCCG-3′.

### Cell culture, colony formation, and cell proliferation assay

RECQ4 KD HEK293 cells generated previously^21^ and parental HEK293 cells (ATCC CRL-1573) were cultured in Dulbecco’s modified Eagle’s medium (DMEM) supplemented with 10% fetal bovine serum (FBS) with streptomycin and penicillin. To generate HEK293 cells stably expressing FLAG-RECQ4 WT or mutant proteins, RECQ4 KD HEK293 cells were transfected with the corresponding pCMV-FLAG-RECQ4 plasmids using lipofectamine 2000 (Invitrogen). Single clones were selected with G418 and analyzed for expression of the FLAG-tagged proteins, with the exception of the RECQ4 fragment analysis shown in Fig. 4d using cells 48 h after transient transfection. For proteasome inhibition, cells were treated with 10 μM MG132 (A2585, APExBIO) for 4 h before harvest. Colony formation assay was carried out by seeding 500 cells per 10 cm dish for a minimum of 14 days. Plates from each experiment were incubated for the same amount of time, stained with 0.5% crystal violet, and dried overnight. For cell irradiation, cells were treated with 6 Gy of IR (Gammacell 3000) and harvested 2 h after IR treatment. The real-time cell growth assay was performed using xCELLigence Real-Time Cell Analysis (RTCA) DP (Agilent). Cell cycle analysis was carried out as previously described^58^. Cells were synchronized at G_2_/M phase using 50 ng/ml nocodazole in complete medium for 20 h. Cells were then released by washing 2x with warm complete DMEM medium. To incorporate BrdU, cells were pulse-labeled with 20 μM BrdU for 30 min before harvest at indicated time points. Cells were then stained with Rat anti-BrdU antibody followed by anti-Rat DyLight 488-conjugated secondary antibody and propidium iodide. Flow cytometry analysis was performed using a CyAn ADP analyser (Beckman Coulter). Cell cycle profile distributions were determined using FlowJo software (Tree Star Inc., OR). An example of ungated vs gated cell profile distribution was included (Supplementary Fig. 5).

### Antibodies

Primary antibodies used were rabbit anti-RECQ4 (4-11) generated against residues 71–80 of human RECQ4 (WB 1:1000)^10^, rabbit anti-RECQ4 (17008-1-AP, Proteintech, IP 1:200), mouse anti-RECQ4 (sc-518189, Santa Cruz, WB 1:1000), mouse anti-alpha tubulin (sc-5286, Santa Cruz, WB 1:1000), rabbit anti-APC1 (21748-1-AP, Proteintech, WB 1:1000), rabbit anti-APC5 (AP7109, Abclonal, WB 1:1000), mouse anti-APC11 (sc-517142, Santa Cruz, WB 1:1000), rabbit anti-beta actin (20536-1-AP, Proteintech, WB 1:1000), rabbit anti-BUB3 (27073-1-AP, Proteintech, WB 1:1000), mouse anti-BUB3 (sc-376506, Santa Cruz, WB 1:1000), rabbit anti-C1QBP (P32) (5734S, Cell Signaling, WB 1:1000), rabbit anti-CDC20 (10252-1-AP, Proteintech, WB 1:1000), rabbit anti-CDC45 (A2047, Abclonal, WB 1:1000), mouse anti-CDC6 (sc-9964, Santa Cruz, WB 1:1000), rabbit anti-CDT1 (A16576, Abclonal, WB 1:1000), rabbit anti-Cyclin A (PA5-16519, Thermo Fisher Scientific, WB 1:1000), mouse anti-Cyclin E (ab3927, Abcam, WB 1:1000), rabbit anti-DNA pol δ (sc-10784, Santa Cruz, WB 1:1000), rabbit anti-FBXO5 (EMI1) (10872-1-AP, Proteintech, WB 1:1000), mouse anti-FLAG (66008-3-Ig, Proteintech, WB 1:1000), rabbit anti-FLAG (20543-1-AP, Proteintech, WB 1:3000), rabbit anti-FZR1 (CDH1) (16368-1-AP, Proteintech, WB 1:1000), rabbit anti-Geminin (10802-1-AP, Proteintech, WB 1:1000), rabbit anti-GINS4 (A8592, Abclonal, WB 1:1000), rabbit anti-Histone H3 (sc-10809, Santa Cruz, WB 1:1000), rabbit anti-Lamin A/C (sc-20681, Santa Cruz, WB 1:1000), rabbit anti-MCM7 (ab52489, Abcam, WB 1:1000), rabbit anti-MCM2 (10513-1-AP, Proteintech, WB 1:1000), mouse anti-MCM5 (sc-165994, Santa Cruz, WB 1:1000), rabbit anti-ORC2 (A302-734A, Bethyl, WB 1:1000), rabbit anti-PSF2 (GINS2) (16247-1-AP, Proteintech, WB 1:1000), mouse anti-GINS2 (sc-376595, Santa Cruz, WB 1:1000), rabbit anti-PP2A (2039S, Cell Signaling, WB 1:1000), rabbit anti-SKP2 (A302-436A, Bethyl, WB 1:1000), rabbit anti-UBE1 (UBA1) (671981-1-Ig, Proteintech, WB 1:1000), rat anti-BrdU/CldU (MCA2060T, Bio-Rad, IF 1:200), mouse anti-BrdU/IdU (347580, BD Biosciences, IF 1:200), goat anti-rat IgG (H + L) Alexa Fluor Plus 488 conjugated (A48262, Invitrogen, IF 1:200), goat anti-mouse IgG (H + L) Alexa Fluor 568 conjugated (A-11004, Invitrogen, IF 1:200), goat anti-rat IgG (H + L) DyLight 488 (SA5-10018, Invitrogen, Flow Cytometry 1:100).

### Lentivirus production and transduction

Lentivirus production was carried out according to the protocol described previously^59^. Briefly, lentiviral particles were produced by co-transfection of HEK293FT cells with 8 μg Tet-pLKO/shAPC5 or Tet-pLKO/Scrambled (Addgene, #47541), 6 μg packaging plasmid psPAX2, and 2 μg envelope plasmid pMD2G by using lipofectamine 2000. The transfection medium was changed after 8 h, and recombinant lentiviruses were harvested 48 h and 72 h later. The virus particles-containing supernatant were centrifuged at 100 × *g* for 5 minutes, filtered through 0.45 μM PES membrane filters, and concentrated using PEG-8000, and aliquoted and stored at −80 °C until needed. For lentivirus transduction, cells were seeded in 10 cm dishes in Tet-free complete DMEM medium. Concentrated virus was directly overlaid on cells and polybrene was added at a final concentration of 10 μg/ml. Medium was changed at 12 h and cells were split at 48 h, then selection was started by adding 2 μg/ml of puromycin for 72 h. shRNA expression was induced by adding doxycycline (20 ng/ml) for 48–72 h.

### Cell fractionation, co-immunoprecipitation, and mass spectrometry

Cell fractionation procedure was adapted from the previously published protocol^10^. Briefly, asynchronized cells in log phase (less than 70% confluency) were harvested and lysed with 3x volume of cytoplasmic lysis buffer (10 mM Tris·HCl pH 7.9, 0.34 M sucrose, 3 mM CaCl_2_, 2 mM magnesium acetate, 0.1 mM EDTA, 1 mM DTT, 0.5% Nonidet P-40, 1x protease inhibitors, and 1x phosphatase inhibitor), and intact nuclei were pelleted by centrifugation at 3500 × *g* for 5 min. Nuclei were washed with cytoplasmic lysis buffer without inhibitors and then lysed with 2x volume of nuclear lysis buffer (20 mM HEPES pH 7.9, 3 mM EDTA, 10% glycerol, 150 mM KCl, 1.5 mM MgCl_2_, 1 mM DTT, 0.1% Nonidet P-40, 1x protease inhibitors, and 1x phosphatase inhibitor) and homogenized using a 21G1/2 needle. The nucleoplasmic fraction was cleared by centrifugation at 15,000 × *g* for 30 min. The chromatin-enriched pellet was then resuspended in 2x volume of nuclease incubation buffer (20 mM Hepes pH 7.9, 1.5 mM MgCl_2_, 150 mM KCl, 10% glycerol, protease inhibitors, and 1x phosphatase inhibitor) and 0.15 unit/μl benzonase (MilliporeSigma). The sample was cleared by centrifugation at 20,000 × *g* for 30 min, and supernatant containing the solubilized native chromatin proteins was collected. Co-immunoprecipitation of FLAG-RECQ4 was conducted using ANTI-FLAG M2 affinity gel (MilliporeSigma). Cell fractions were incubated overnight with ANTI-FLAG M2 affinity gel at 4 °C. Beads were pelleted by centrifugation and washed extensively with FLAG-A-binding buffer (10 mM HEPES pH 7.9, 1.5 mM MgCl_2_, 0.3 M NaCl, 10 mM KCl, 0.2% Triton X-100, and 10% glycerol). The FLAG-tagged protein complexes were eluted by using either SDS loading buffer or FLAG elution A buffer (20 mM HEPES 7.9, 0.2 M NaCl, 0.2 mM EDTA, 0.05% Triton-X, 0.3 mg/ml 3xFLAG peptide, and 10% glycerol). For mass spectrometry analysis, purified FLAG-RECQ4 protein complexes were loaded onto a Mini-PROTEAN precast polyacrylamide gel (BioRad). After the eluent migrated 1 cm from the well into the gel, the gel was fixed with collodial coomassie stain (Invitrogen), and the 1 cm gel slice was excised and stored in ddH2O prior to mass spectrometry analysis. Mass spectrometry analysis was performed by the Taplin Biological Mass Spectrometry Core Facility at Harvard University^60–62^. Briefly, gel slices were resuspended in reducing buffer (50 mM NH_4_HCO_3_, 1 mM DTT) for 30 min at 60 °C, followed by the addition of 5 mM iodoacetamide for 15 min at room temperature and quenching with 5 mM DTT. Gel slices were washed, dehydrated with acetonitrile, and dried. For trypsin digestion, gel slices were rehydrated in 50 mM NH_4_HCO_3_ containing 12.5 ng/μl modified sequencing-grade trypsin (Promega, V5111) and then incubated at 37 °C overnight. Peptides were extracted and reconstituted in 10 µl of solvent A (2.5% acetonitrile, 0.1% formic acid). Each digested sample was loaded via a Famos autosampler (LC Packings) onto a nano-scale reverse-phase HPLC capillary column, which was prepared by packing 2.6 µm C18 spherical silica beads into a fused silica capillary (100 µm inner diameter; 30 cm length). Peptides were eluted with increasing concentrations of solvent B (97.5% acetonitrile, 0.1% formic acid). Peptides, as they were eluted from the column, were subjected to electrospray ionization before entering an LTQ Orbitrap Velos Pro ion-trap mass spectrometer (Thermo Fisher Scientific) to produce a tandem mass spectrum of specific fragment ions for each peptide. Software program Sequest (Thermo Fisher Scientific) was used to determine peptide sequences and protein identity by matching the acquired fragmentation pattern with the human proteome database. All databases include a reversed version of all the sequences and the data was filtered with a cutoff at 1% peptide false discovery rate. Endogenous RECQ4 and associated proteins were immunopurified from parental HEK293 WCE prepared from the indicated time points post nocodazole release using a rabbit anti-RECQ4 antibody (Proteintech) conjugated to protein A agarose beads (Pierce).

### DNA fiber analysis

Cells were labeled with 50 μM IdU for 30 min and then 50 μM CldU for 30 min. Cells were then trypsinized and resuspended in ice-cold PBS at 2.5 × 10^5^ cell/ml. DNA spreads were made as previously described^63^ with modifications. Briefly, 2 μl cell suspensions were mixed with 8 μl spreading buffer (200 mM Tris-HCl pH 7.4, 50 mM EDTA, 0.5% SDS) on a silane-coated slide. After 5 min, the slides were tilted at 15° to allow DNA to run down the slide, air dried, and then fixed in methanol/acetic acid (3:1). The slides were treated with 2.5 N HCl at 37 °C for 1 h, washed 3 times in PBS, and blocked with 2% bovine serum albumin in PBS. The slides were then incubated with mouse anti-BrdU/IdU and rat anti-BrdU/CldU in blocking buffer at 4 °C overnight. After washing, the slides were incubated in secondary antibody mixture of Alexa Fluor 488-conjugated goat anti-rat and Alexa Fluor 568-conjugated goat anti-mouse (Invitrogen) at room temperature for 1 h. The slides were then washed and mounted in Prolong gold antifade (Invitrogen). Microscopy was carried out using a Zeiss Axio Observer with oil immersion lens. Fiber length was measured based on a conversion factor of 1 µm to 2.59 kb.

### Protein expression and purification

Recombinant His-tagged, FLAG-tagged RECQ4 WT, and mutant fragments were prepared as previously described^64^. Ubiquitin was overexpressed in *E. coli* BL21(DE3) (Novagen) and induced by 0.8 mM IPTG for 4 h at 30 °C in LB medium. After cell lysis and centrifugation, Ubiquitin was affinity purified from supernatant using HisPur Cobalt resin (Thermo Fisher Scientific). Geminin was overexpressed in *E. coli* BL21(DE3) and induced by 0.1 mM IPTG overnight at 16 °C in LB medium. After cell lysis and centrifugation, supernatant was loaded onto chitin resin (NEB). Geminin was cleaved and eluted from chitin resin with 50 mM DTT. All proteins were dialysis against 20 mM Tris pH 7.5, 150 mM NaCl, 0.1 mM EDTA.

### In vitro DNA binding, protein pull-down, and ubiquitination assays

DNA binding reactions, which were based on the previously published protocol^64^, were initiated by incubating WT or mutant RECQ4 proteins with 4 pg of ^32^P-labeled single-stranded DNA oligo in DNA binding buffer (30 mM HEPES pH 7.5, 1 mM DTT, 100 µg/ml BSA) on ice for 15 min. The reactions were cross-linked with 1% glutaraldehyde at 37 °C for 15 min, and the protein-DNA complexes were analyzed on 5% native polyacrylamide gels. For APC/C pull-down from the chromatin fraction using purified recombinant His-RECQ4-FLAG protein fragments, CB fractions were incubated with M2 agarose pre-bound with His-RECQ4-FLAG protein fragments for 4 h at 4 °C. Unbound proteins were removed by washing the beads extensively with FLAG-A-binding buffer. For in vitro ubiquitination assays, cell lysates were prepared from RECQ4 KD HEK293 cells stably overexpressing FLAG-RECQ4 WT or Q757X mutant. To deplete APC/C complexes in cell lysates, 2 µg APC5 antibody or rabbit IgG (control) was added into 100 µl each cell lysate and incubated overnight at 4 °C, and protein A/G beads (Santa Cruz) were used to immunoprecipitate APC/C complexes. Geminin was labeled with Fluorescein isothiocyanate (FITC) by incubating 2 µg Geminin with 100 ng FITC for 2 h at room temperature, then dialyzing against 20 mM Tris pH 7.5, 150 mM NaCl, 0.1 mM EDTA. Ubiquitination assay was carried out by mixing various concentrations of cell lysates with 500 nM FITC-Geminin, 100 nM UBA1 (R&D SYSTEM, catalog: E-305), 250 nM Ube2S (G-Bioscience, catalog: BAN0353), 250 nM Ube2C (R&D SYSTEM, catalog: E2-654), 25 nM CDH1 (AVIVA Systems Biology, catalog: OPCA02549), 60 µM Ubiquitin, and 1 µM ubiquitin aldehyde (AdipoGen Life Sciences, catalog: SBB-PS0031) in reaction buffer (40 mM Tris-HCl (pH 7.5), 1 mM DTT, 5 mM MgCl_2_, 3 mM ATP) with or without 0.1% SDS in total 10 μl reaction volume for 1 h at 37 ˚C. The extent of ubiquitination of Geminin was resolved by 10% SDS-PAGE and visualized by Odyssey Fc (LI-COR) at 600 nm.

### Statistics and reproducibility

For all experiments, unless stated otherwise, representative analyses from a minimum of three independent experiments with similar results are shown. All in vitro analyses using purified recombinant proteins were performed at least three times using two different sets of purified proteins. For cell growth analyses, each value represents mean ± standard deviation (SD) calculated from a minimum of three biologically independent samples. *p* values were calculated using two-tailed student’s t-tests for statistically significant differences.

### Reporting summary

Further information on research design is available in the Nature Portfolio Reporting Summary linked to this article.

## Supplementary information

Supplementary Information

Description of Additional Supplementary Files

Supplementary Data 1

Supplementary Data 2

Reporting Summary

## Data Availability

All data generated or analyzed during this study are included in this published article (and its Supplementary Information files). The full list of mass spectrometry data generated in this study are provided in Supplementary Data 1 and 2. The mass spectrometry proteomics data generated in this study have been deposited to the ProteomeXchange Consortium via the MassIVE partner repository with the dataset identifier PXD040394 for FLAG-RECQ4 WT and PXD040395 for Q757X chromatin-bound complexes. Source data are provided with this paper.

## Source data

Source Data
