# Supplementary material for: DNA replication initiation factor RECQ4 possesses a role in antagonizing DNA replication initiation
Source: Nat Commun. 2023 Mar 4;14:1233. doi: 10.1038/s41467-023-36968-1 (PMC9985596; doi:10.1038/s41467-023-36968-1)
Supplement: Supplementary file 1 — Supplementary Information [file 41467_2023_36968_MOESM1_ESM.pdf]

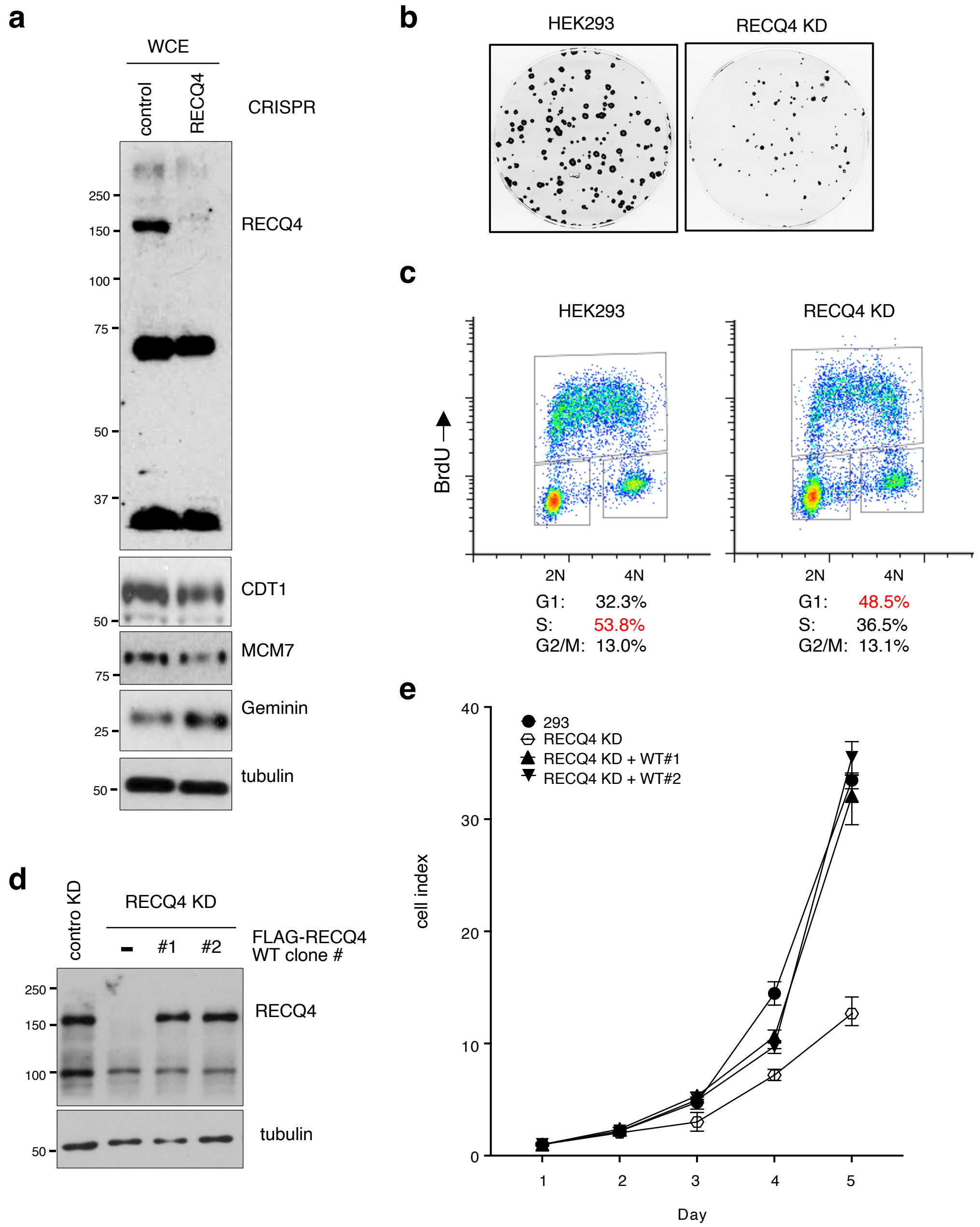

Supplementary Figure 1. Analysis of RECQ4 KD cells. (a) Representative western blot analysis of the indicated proteins in whole-cell extracts (WCE) prepared from HEK293 control or CRISPR-edited RECQ4 knockdown (KD) cells. Tubulin was used as a loading control. Source data are provided as a Source Data file. (b) Representative colony formation assay to measure growth rate of HEK293 control and RECQ4 KD cells at Day 18 after seeding. (c) Representative flow cytometry analysis of HEK293 control and RECQ4 KD cells. The cells were labeled with BrdU and co-stained for BrdU incorporation (Y-axis) and DNA content (propidium iodide [PI], X-axis). Percentages of cells in G_1, S, and G_2/M phases are shown, with the highest percentage in each time point shown in red. (d) Representative western blot analysis of RECQ4 expression in WCE prepared from HEK293 control, RECQ4 KD, or RECQ4 KD cells stably expressing wildtype (WT) FLAG-RECQ4. Tubulin was used as a loading control. Source data are provided as a Source Data file. (e) Representative real-time cell growth assay to measure cell growth rate of HEK293 control, RECQ4 KD, or RECQ4 KD cells stably expressing WT FLAG-RECQ4. Source data are provided as a Source Data file. n = 3 (biologically independent samples). Data are presented as mean values ± SD.

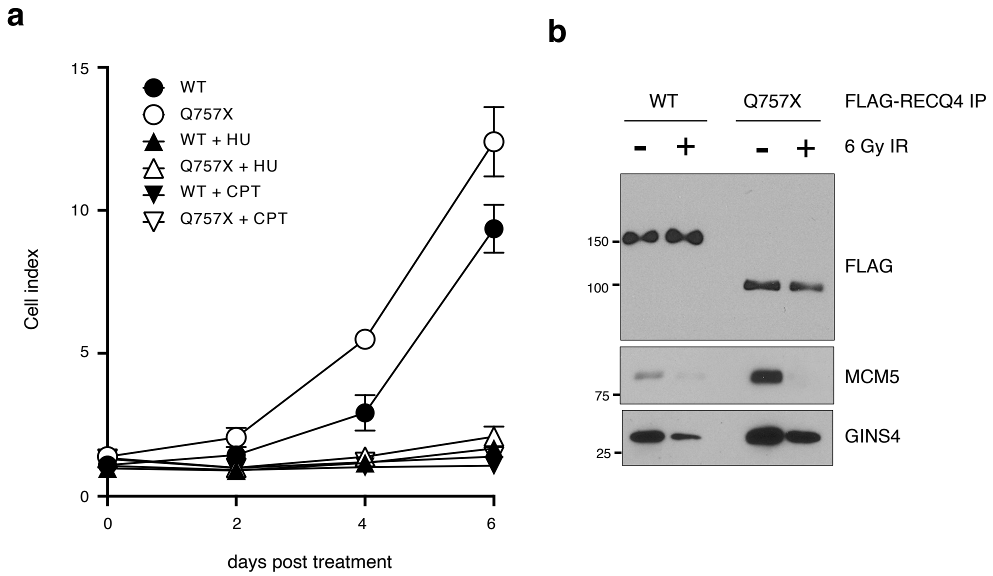

Supplementary Figure 2. FLAG-RECQ4 WT and Q757X mutant complexes in response to DNA damage. (a) Representative real-time cell growth assays to measure cell growth rate of RECQ4 knockdown (KD) HEK293 cells stably expressing FLAG-tagged RECQ4 wildtype (WT) or Q757X mutant proteins and treated with 1 mM hydroxyurea (HU) or 100 nM camptothecin (CPT). Source data are provided as a Source Data file. n = 3 (biologically independent samples). Data are presented as mean values ± SD. (b) Representative western blot analysis of the indicated proteins co-purified with FLAG-RECQ4 WT or Q757X proteins from chromatin-bound (CB) fractions prepared from RECQ4 WT and Q757X expressing cells 2 h after the cells were treated with or without 6 Gy of ionizing radiation (IR). IP = immunoprecipitation. Source data are provided as a Source Data file.

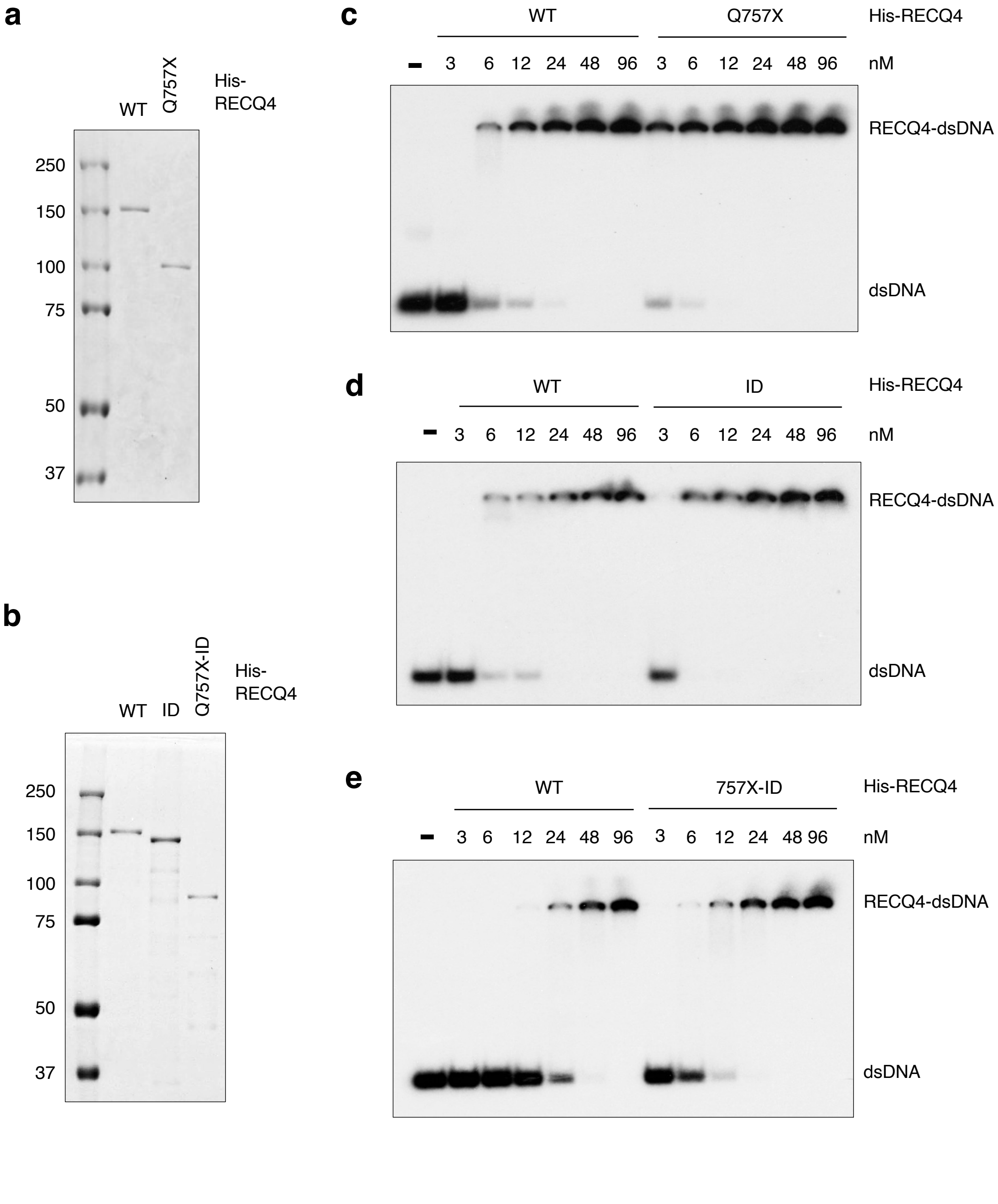

Supplementary Figure 3. Protein-protein and protein-DNA interactions of the RECQ4 mutants. (a-b) Recombinant N-terminal His-tagged and C-terminal FLAG-tagged RECQ4 WT and indicated mutant proteins were overexpressed and purified from *E. coli*, separated by SDS-PAGE, and stained with Coomassie blue. (c-e) Representative images of electrophoretic mobility shift assays of His-FLAG-RECQ4 Q757X (c), internal deletion (ID, d), and Q757X-ID (e) proteins binding to ^{32}P end-labeled dsRNA oligo substrate compared to WT RECQ4.

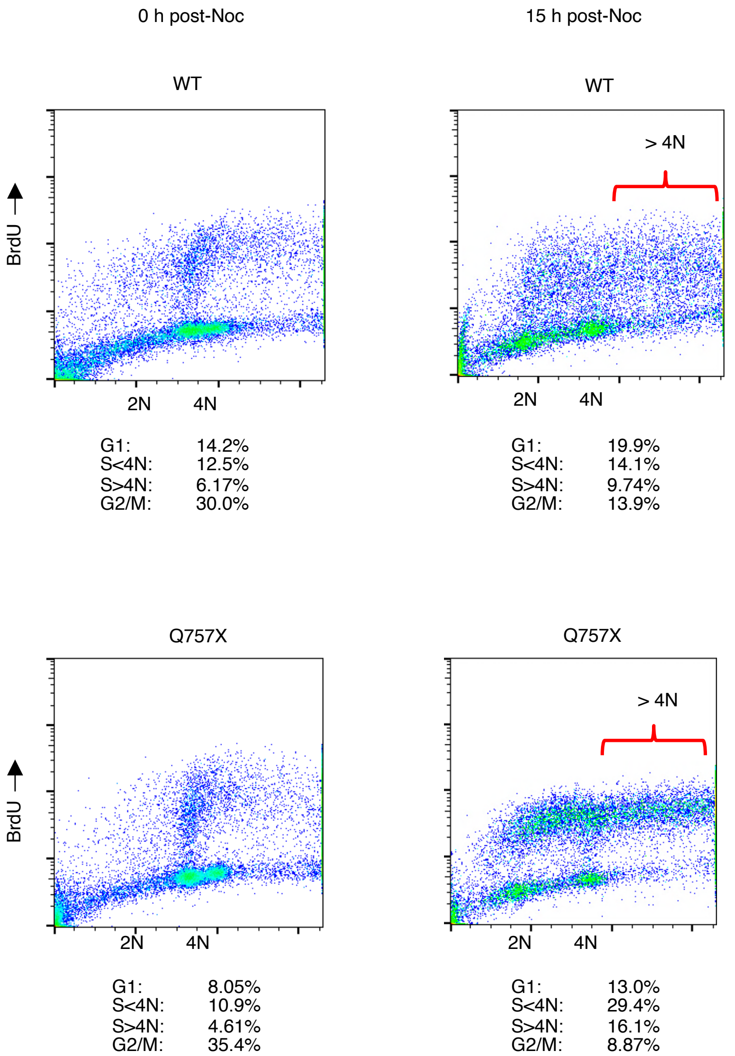

Supplementary Figure 4. Increasing DNA contents in the RECQ4 Q757X mutant cells. Representative flow cytometry analysis of RECQ4 knockdown (KD) HEK293 cells stably expressing FLAG-RECQ4 wildtype (WT) or Q757X mutant in each indicated time point after nocodazole (Noc) release. Percentages of cells in G_1, S, and G_2/M phases are shown. Cells with >4N DNA content are labeled.

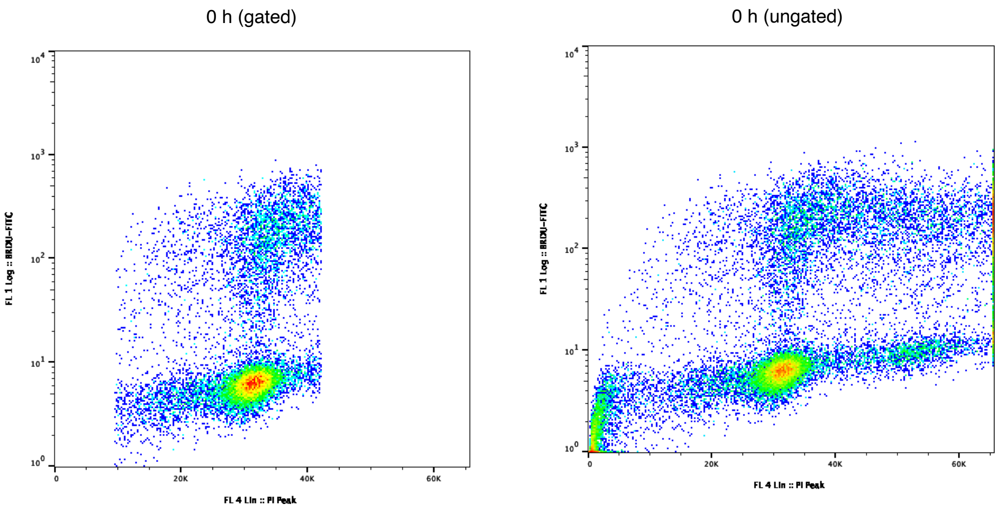

Supplementary Figure 5. Example of the gated and ungated flow cytometry analysis Example of the gated (left) and ungated (right) flow cytometry analysis of the parental HEK293 cells synchronized to 0 hour (h) post nocodazole release as shown in Fig. 3a.

Supplementary References

1. Xu, X., Rochette, P.J., Feyissa, E.A., Su, T.V. & Liu, Y. MCM10 mediates RECQ4 association with MCM2-7 helicase complex during DNA replication. *EMBO J* **28**, 3005-14 (2009).
