# Supplementary material for: DNA replication initiation factor RECQ4 possesses a role in antagonizing DNA replication initiation
Source: Nat Commun. 2023 Mar 4;14:1233. doi: 10.1038/s41467-023-36968-1 (PMC9985596; doi:10.1038/s41467-023-36968-1)
Supplement: Supplementary file 2 — Description of Additional Supplementary Files [file 41467_2023_36968_MOESM2_ESM.pdf]

Description of Additional Supplementary Files

File Name: Supplementary Data 1
Description: **Mass spectrometry analysis of chromatin-bound (CB) FLAG-RECQ4 wildtype (WT) and Q757X (Q7) protein complexes.** FLAG-RECQ4 complexes were immunopurified from CB fractions prepared from RECQ4 KD HEK293 cells stably expressing FLAG-RECQ4 WT or Q757X mutant constructs. Purified complexes were subjected to mass spectrometry analysis. n = 1 (biologically independent samples).

File Name: Supplementary Data 2
Description: **Additional mass spec data set of the chromatin-bound (CB) FLAG-RECQ4. The** full list of the proteins identified the FLAG-RECQ4 complexes purified from the CB fraction of HEK293T cells stably expressing FLAG-RECQ4 from the previous study 1 . The replication factors MCM10, MCM2-7, CDC45, GINS, TIMELESS and TIPIN were previously reported 1 . n = 1 (biologically independent samples).
