# Supplementary material for: DNA replication initiation factor RECQ4 possesses a role in antagonizing DNA replication initiation
Source: Nat Commun. 2023 Mar 4;14:1233. doi: 10.1038/s41467-023-36968-1 (PMC9985596; doi:10.1038/s41467-023-36968-1)
Supplement: Supplementary file 5 — Reporting Summary [file 41467_2023_36968_MOESM5_ESM.pdf]

nature portfolio

Corresponding author(s): Yilun Liu

Last updated by author(s): February 16, 2023

Reporting Summary

Nature Portfolio wishes to improve the reproducibility of the work that we publish. This form provides structure for consistency and transparency in reporting. For further information on Nature Portfolio policies, see our Editorial Policies and the Editorial Policy Checklist.

Please do not complete any field with "not applicable" or n/a. Refer to the help text for what text to use if an item is not relevant to your study. For final submission: please carefully check your responses for accuracy; you will not be able to make changes later.

Statistics

For all statistical analyses, confirm that the following items are present in the figure legend, table legend, main text, or Methods section.

n/a	Confirmed	
☐	☒	The exact sample size (*n*) for each experimental group/condition, given as a discrete number and unit of measurement
☐	☒	A statement on whether measurements were taken from distinct samples or whether the same sample was measured repeatedly
☐	☒	The statistical test(s) used AND whether they are one- or two-sided<br>*Only common tests should be described solely by name; describe more complex techniques in the Methods section.*
☐	☒	A description of all covariates tested
☐	☒	A description of any assumptions or corrections, such as tests of normality and adjustment for multiple comparisons
☐	☒	A full description of the statistical parameters including central tendency (e.g. means) or other basic estimates (e.g. regression coefficient) AND variation (e.g. standard deviation) or associated estimates of uncertainty (e.g. confidence intervals)
☐	☒	For null hypothesis testing, the test statistic (e.g. *F*, *t*, *r*) with confidence intervals, effect sizes, degrees of freedom and *P* value noted<br>*Give P values as exact values whenever suitable.*
☒	☐	For Bayesian analysis, information on the choice of priors and Markov chain Monte Carlo settings
☒	☐	For hierarchical and complex designs, identification of the appropriate level for tests and full reporting of outcomes
☒	☐	Estimates of effect sizes (e.g. Cohen's *d*, Pearson's *r*), indicating how they were calculated

Our web collection on statistics for biologists contains articles on many of the points above.

Software and code

Policy information about availability of computer code

Data collection	No software was used for data collection
Data analysis	FlowJo OS 10.6.1 for all cell cycle analyses. ImageJ OS 1.52 for western blot quantifications for Source Data. Sequest by Harvard Taplin Mass Spectrometry Facility

For manuscripts utilizing custom algorithms or software that are central to the research but not yet described in published literature, software must be made available to editors and reviewers. We strongly encourage code deposition in a community repository (e.g. GitHub). See the Nature Portfolio guidelines for submitting code & software for further information.

Data

Policy information about availability of data

All manuscripts must include a data availability statement. This statement should provide the following information, where applicable:

- Accession codes, unique identifiers, or web links for publicly available datasets
- A description of any restrictions on data availability
- For clinical datasets or third party data, please ensure that the statement adheres to our policy

Data Availability section is included under Methods. All data generated or analyzed during this study are included in this published article (and its supplementary information files). The raw mass spectrometry data used in this study to compare FLAG-RECQ4 wildtype and Q757X chromatin-bound complexes in Supplementary

Data 1 are available in the MassIVE under [ftp://massive.ucsd.edu/MSV000088652 for WT and ftp://massive.ucsd.edu/MSV000088653/ for Q757x. These links are also included in Data Availability section under Methods.

Human research participants

Policy information about studies involving human research participants and Sex and Gender in Research.

Reporting on sex and gender	N/A
Population characteristics	N/A
Recruitment	N/A
Ethics oversight	N/A

Note that full information on the approval of the study protocol must also be provided in the manuscript.

Field-specific reporting

Please select the one below that is the best fit for your research. If you are not sure, read the appropriate sections before making your selection.

☒ Life sciences ☐ Behavioural & social sciences ☐ Ecological, evolutionary & environmental sciences

Life sciences study design

All studies must disclose on these points even when the disclosure is negative.

Sample size	For DNA fiber analysis, at least 150 DNA fibers were analyzed and scored per standard published protocol (PMID:32584505). For colony formation assays, 500 cells were plated per plate and 3 biological independent samples were analyzed per experiment. For real time cell analysis, a minimum of 3 biologically independent samples per cell line were included per experiment to establish p value. For flow cytometry analyses, approximately 2x10^4 cells were used in each time point and gating was applied to cells with 4N or less DNA content, except Supplementary Figure 4. For protein complex purifications and western blot analysis, approximately 10^7 cells were harvested and fractionated per samples.
Data exclusions	No data were excluded from the analyses
Replication	For all experiments, unless stated otherwise, representative analyses from a minimum of three independent experiments are shown. All in vitro analyses using purified recombinant proteins were performed at least three times using 2 different sets of purified proteins. All experiments were successfully replicated. Key conclusions were also validated by multiple techniques. For example, changes in protein-protein interactions were validated by mass spec, western blots and in vitro biochemical pull down assay performed by different authors. Cell growth changes were validated by colony formation, real-time growth assays and cell cycle progression. For cell growth analyses, each value represents mean ± standard deviation calculated from 3 independent biological samples for one representative experiment. p values were calculated using two-tailed student's t-tests for statistically significant differences.
Randomization	Randomization was not applicable for all cell based assays (immunoprecipitation, fractionations, DNA fiber analysis, cell cycle, cell growth rate) to compare biochemical and cellular functions of the control and the defined mutant cell lines. Randomization was also not applicable for in vitro biochemical assays using purified recombinant proteins
Blinding	Blinding was not applicable for experiments that involved cell fractionation, protein complex purification and protein-protein interaction assays and in vitro biochemical assays. Instead, key experiments were repeated by different authors to ensure reproducibility.

Reporting for specific materials, systems and methods

We require information from authors about some types of materials, experimental systems and methods used in many studies. Here, indicate whether each material, system or method listed is relevant to your study. If you are not sure if a list item applies to your research, read the appropriate section before selecting a response.

Materials & experimental systems

n/a	Involved in the study
☐	☒ Antibodies
☐	☒ Eukaryotic cell lines
☒	☐ Palaeontology and archaeology
☒	☐ Animals and other organisms
☒	☐ Clinical data
☒	☐ Dual use research of concern

Methods

n/a	Involved in the study
☒	☐ ChIP-seq
☐	☒ Flow cytometry
☒	☐ MRI-based neuroimaging

Antibodies

Antibodies used

Primary antibodies used were rabbit anti-RECQ4 (4-11) generated against residues 71-80 of human RECQ4 (WB 1:1000), rabbit anti-RECQ4 (17008-1-AP, Proteintech, IP 1:200), mouse anti-RECQ4 (sc-518189, Santa Cruz, WB 1:1000), mouse anti-alpha tubulin (sc-5286, Santa Cruz, WB 1:1000), rabbit anti-APC1 (21748-1-AP, Proteintech, WB 1:1000), rabbit anti-APC5 (AP7109, Abclonal, WB 1:1000), mouse anti-APC11 (sc-517142, Santa Cruz, WB 1:1000), rabbit anti-beta actin (20536-1-AP, Proteintech, WB 1:1000), rabbit anti-BUB3 (27073-1-AP, Proteintech, WB 1:1000), mouse anti-BUB3 (sc-376506, Santa Cruz, WB 1:1000), rabbit anti-C1QBP (P32) (5734S, Cell Signaling, WB 1:1000), rabbit anti-CDC20 (10252-1-AP, Proteintech, WB 1:1000), rabbit anti-CDC45 (A2047, Abclonal, WB 1:1000), mouse anti-CDC6 (sc-9964, Santa Cruz, WB 1:1000), rabbit anti-CDT1 (A16576, Abclonal, WB 1:1000), rabbit anti-Cyclin A (PA5-16519, ThermoFisher Scientific, WB 1:1000), mouse anti-Cyclin E (ab3927, Abcam, WB 1:1000), rabbit anti-DNA pol δ (sc-10784, Santa Cruz, WB 1:1000), rabbit anti-FBXO5 (EMI1) (10872-1-AP, Proteintech, WB 1:1000), mouse anti-FLAG (66008-3-Ig, Proteintech, WB 1:1000), rabbit anti-FLAG (20543-1-AP, Proteintech, WB 1:3000), rabbit anti-FZR1 (CDH1) (16368-1-AP, Proteintech, WB 1:1000), rabbit anti-Geminin (10802-1-AP, Proteintech, WB 1:1000), rabbit anti-GINS4 (A8592, Abclonal, WB 1:1000), rabbit anti-Histone H3 (sc-10809, Santa Cruz, WB 1:1000), rabbit anti-Lamin A/C (sc-20681, Santa Cruz, WB 1:1000), rabbit anti-MCM7 (ab52489, Abcam, WB 1:1000), rabbit anti-MCM2 (10513-1-AP, Proteintech, WB 1:1000), mouse anti-MCM5 (sc-165994, Santa Cruz, WB 1:1000), rabbit anti-ORC2 (A302-734A, Bethyl, WB 1:1000), rabbit anti-PSF2 (GINS2) (16247-1-AP, Proteintech, WB 1:1000), mouse anti-GINS2 (sc-376595, Santa Cruz, WB 1:1000), rabbit anti-PP2A (2039S, Cell Signaling, WB 1:1000), rabbit anti-SKP2 (A302-436A, Bethyl, WB 1:1000), rabbit anti-UBE1 (UBA1) (671981-1-Ig, Proteintech, WB 1:1000), rat anti-BrdU/CldU (MCA2060T, Bio-Rad, IF 1:200), mouse anti-BrdU/IdU (347580, BD Biosciences, IF 1:200), goat anti-rat IgG (H+L) Alexa Fluor Plus 488 conjugated (A48262, Invitrogen, IF 1:200), goat anti-mouse IgG (H+L) Alexa Fluor 568 conjugated (A-11004, Invitrogen, IF 1:200), goat anti-rat IgG (H+L) DyLight 488 (SA5-10018, Invitrogen, Flow Cytometry 1:100).

Validation

RECQ4 and APC5 antibodies were validated by western blots using RECQ4 and APC5 KD cells. Commercially validated antibodies are:
1. rabbit anti-RECQ4: https://www.ptglab.com/products/RECQL4-Antibody-17008-1-AP.htm
"17008-1-AP targets RECQL4 in WB, IP, IHC, IF, ELISA applications and shows reactivity with human samples."
2. mouse anti-RECQ4: https://www.scbt.com/p/recql4-antibody-b-3
"recommended for detection of RecQL4 of mouse, rat and human origin by WB, IP, IF and ELISA"
3. mouse anti-alpha tubulin - https://www.scbt.com/p/alpha-tubulin-antibody-b-7?gclid=EAIaIQobChMIxeey497h_AIVMSc4Ch2aiQOUEAAYASAAEgK3evD_BwE
"detection of α Tubulin of mouse, rat and human origin by WB, IP, IF, IHC(P), FCM and ELISA; also reactive with additional species, including and canine, bovine and porcine"
4. rabbit anti-APC1: https://www.ptglab.com/products/ANAPC1-Antibody-21748-1-AP.htm
"Positive WB detected in HeLa cells, HEK-293 cells, HT-1080 cells, K-562 cells, mouse brain tissue, Transfected HEK-293 cells"
5. rabbit anti-APC5: https://abclonal.com/catalog-antibodies/KOValidatedANAPC5RabbitpAb/A7109
"KO Validated"
6. mouse anti-APC11: https://www.scbt.com/p/apc11-antibody-1b4-1a4
"recommended for detection of APC11 of human origin by WB, IP and ELISA"
7. rabbit anti-beta actin: https://www.ptglab.com/products/ACTB-Antibody-20536-1-AP.htm
"KD/KO validated"
8. rabbit anti-BUB3: https://www.ptglab.com/products/BUB3-Antibody-27073-1-AP.htm
"Positive WB detected in HEK-293T cells, C6 cells"
9. mouse anti-BUB3: https://www.scbt.com/p/bub3-antibody-e-7
"recommended for detection of BUB3 of mouse, rat and human origin by WB, IP, IF and ELISA; also reactive with additional species, including and equine, canine and porcine"
10. rabbit anti-C1QBP: https://www.cellsignal.com/products/primary-antibodies/c1qbp-antibody/5734
"C1QBP Antibody recognizes endogenous levels of total C1QBP protein."
11. anti-CDC20: https://www.ptglab.com/products/CDC20-Antibody-10252-1-AP.htm
"Positive WB detected in HEK-293 cells, HeLa cells, PC-3 cells, HL-60 cells, Jurkat cells, HepG2 cells"
12. rabbit anti-CDC45: https://abclonal.com/catalog-antibodies/CDC45PolyclonalAntibody/A2047
"tested application - WB"
13. mouse anti-CDC6: https://www.scbt.com/p/cdc6-antibody-180-2
"recommended for detection of Cdc6 of mouse, rat and human origin by WB, IP, IF and IHC(P)"
14. rabbit anti-CDT1: https://abclonal.com/catalog-antibodies/CDT1RabbitpAb/A16576
"tested application - WB"
15. rabbit anti-Cyclin A: https://www.thermofisher.com/antibody/product/Cyclin-A-Antibody-Polyclonal/PA5-16519
" PA5-16519 targets Cyclin A in IHC (P), IP, and WB applications and shows reactivity with mouse, Rat, and Human samples "
16. mouse anti-Cyclin E: https://www.citeab.com/antibodies/725685-ab3927-anti-cyclin-e1-antibody-he12
"validations - knockout", product discontinued
17. rabbit anti-DNA pol δ: https://www.scbt.com/p/dna-pol-delta-cat-antibody-h-300, product discontinued
18. rabbit anti-FBXO5 (EMI1): https://www.ptglab.com/products/FBXO5-Antibody-10872-1-AP.htm
"Positive WB detected in HEK-293 cells, HeLa cells, HepG2 cells, human ovary tissue, K-562 cells, mouse kidney tissue, rat liver

tissue"
19. mouse anti-FLAG: https://www.ptglab.com/products/Flag-tag-Antibody-66008-3-Ig.htm
" 66008-3-Ig targets DYKDDDDK tag in WB, RIP, IP, IHC, IF, CoIP, ELISA applications and shows reactivity with recombinant protein samples."
20. rabbit anti-FLAG: https://www.ptglab.com/products/Flag-Tag-Antibody-20543-1-AP.htm
" Tested Reactivity recombinant protein"
21. rabbit anti-FZR1 (CDH1): https://www.ptglab.com/products/FZR1-Antibody-16368-1-AP.htm
"Positive WB detected in HeLa cells, C6 cells, HepG2 cells, Jurkat cells, mouse heart tissue, mouse liver tissue, NIH/3T3 cells"
22. rabbit anti-Geminin: https://www.ptglab.com/products/GMNN-Antibody-10802-1-AP.htm
" Positive WB detected in HEK-293 cells, human testis tissue, mouse testis tissue, HeLa cells; mouse testis tissue, rat testis tissue"
23. rabbit anti-GINS4: https://abclonal.com/catalog-antibodies/GINS4RabbitpAb/A8592
"tested application - WB"
24. rabbit anti-Histone H3: https://www.scbt.com/p/histone-h3-antibody-fl-136, product discontinued
25. rabbit anti-Lamin A/C: https://www.scbt.com/p/lamin-a-c-antibody-h-110, product discontinued
26. rabbit anti-MCM7: https://www.abcam.com/mcm7prl-antibody-ep1974y-ab52489.html
" Suitable for: Flow Cyt (Intra), ICC/IF, WB, IP, IHC-P"
27. rabbit anti-MCM2: https://www.ptglab.com/products/MCM2-Antibody-10513-1-AP.htm
"Positive WB detected in HEK-293 cells, HeLa cells, PC-3 cells, K-562 cells"
28. mouse anti-MCM5: https://www.scbt.com/p/mcm5-antibody-e-10
" recommended for detection of MCM5 of mouse, rat and human origin by WB, IP, IF, IHC(P) and ELISA"
29. rabbit anti-ORC2: https://www.thermofisher.com/antibody/product/ORC2-Antibody-Polyclonal/A302-734A
"applications WB, IHC, IP"
30. rabbit anti-PSF2 (GINS2): https://www.ptglab.com/products/GINS2-Antibody-16247-1-AP.htm
"Positive WB detected in Jurkat cells, HeLa cells, HepG2 cells"
31. mouse anti-GINS2: https://www.scbt.com/p/psf2-antibody-f-7
" recommended for detection of Psf2 of mouse, rat and human origin by WB, IP, IF and ELISA"
32. rabbit anti-PP2A: https://www.cellsignal.com/products/primary-antibodies/pp2a-a-subunit-antibody/2039
"This antibody detects endogenous levels of PP2A A subunit, alpha isoform. The antibody does not cross-react with other PP2A subunits."
33. rabbit anti-SKP2: https://www.thermofisher.com/antibody/product/SKP2-Antibody-Polyclonal/A302-436A
"applications WB, IP"
34. rabbit anti-UBE1 (UBA1): https://www.ptglab.com/products/UBE1-Antibody-67198-1-Ig.htm
"67198-1-Ig targets UBE1 in WB, IHC, IF applications and shows reactivity with Human, mouse, rat samples."
35. rat anti-BrdU/CldU: https://www.citeab.com/antibodies/115703-mca2060t-rat-anti-brdu, product discontinued
36. mouse anti-BrdU/IdU: https://www.bdbiosciences.com/en-us/products/reagents/flow-cytometry-reagents/clinical-discovery-research/single-color-antibodies-ruo-gmp/purified-mouse-anti-brdu.347580
" Application: Flow cytometry, Intracellular staining (flow cytometry)"
37. goat anti-rat IgG (H+L) Alexa Fluor Plus 488 conjugated: https://www.thermofisher.com/antibody/product/Goat-anti-Rat-IgG-H-L-Highly-Cross-Adsorbed-Secondary-Antibody-Polyclonal/A48262
"these goat anti-mouse IgG (H+L) whole secondary antibodies have been affinity purified and cross-adsorbed against human IgG and human serum prior to conjugation. "
38. goat anti-mouse IgG (H+L) Alexa Fluor 568 conjugated: https://www.thermofisher.com/antibody/product/Goat-anti-Mouse-IgG-H-L-Cross-Adsorbed-Secondary-Antibody-Polyclonal/A-11004
"these goat anti-mouse IgG (H+L) whole secondary antibodies have been affinity purified and cross-adsorbed against human IgG and human serum prior to conjugation"
39. goat anti-rat IgG (H+L) DyLight 488: https://www.thermofisher.com/antibody/product/Goat-anti-Rat-IgG-H-L-Cross-Adsorbed-Secondary-Antibody-Polyclonal/SA5-10018

Eukaryotic cell lines

Policy information about cell lines and Sex and Gender in Research

Cell line source(s)	HEK293 from ATCC
Authentication	Primers specific to the human RECQ4 gene were used to PCR and sequence the RECQ4 gene before and after CRISPR editing of the parental HEK293 cells. The parental HEK293 cell line was also sequenced using primers specific for human topoisomerase I and RECQ5. All the RECQ4 mutant cell lines derived from the parental cell lines were authenticated by sequencing using primers specific to human RECQ4 and western blot analyses using rabbit RECQ4 antibody specific to human RECQ4 residues 71-80.
Mycoplasma contamination	All cell lines generated in this study have been tested negative for mycoplasma
Commonly misidentified lines (See ICLAC register)	No commonly misidentified cell lines were used.

Flow Cytometry

Plots

Confirm that:

☒ The axis labels state the marker and fluorochrome used (e.g. CD4-FITC).

☒ The axis scales are clearly visible. Include numbers along axes only for bottom left plot of group (a 'group' is an analysis of identical markers).

☒ All plots are contour plots with outliers or pseudocolor plots.

☒ A numerical value for number of cells or percentage (with statistics) is provided.

Methodology

Sample preparation	Cells were synchronized at G2/M phase using 50 ng/ml nocodazole in complete medium for 20 h. Cells were then released by washing 2x with warm complete DMEM medium. To incorporate BrdU, cells were pulse-labelled with 20 µM BrdU for 30 min before harvest at indicated time points. Cells were then stained with Rat anti-BrdU antibody followed by anti-Rat Alexa 488-conjugated secondary antibody and propidium iodide.
Instrument	Flow cytometry analysis was performed using a CyAn ADP analyser (Beckman Coulter).
Software	Cell cycle profile distributions were determined using FlowJo software (Tree Star Inc., OR).
Cell population abundance	Percentages of cells in different cell cycle stages are quantified using FlowJo and presented in the corresponding figures
Gating strategy	All flow cytometry graphs except Supplementary Fig 4 are gated to show those cells with 2N < DNA < 4N

☒ Tick this box to confirm that a figure exemplifying the gating strategy is provided in the Supplementary Information.

This checklist template is licensed under a Creative Commons Attribution 4.0 International License, which permits use, sharing, adaptation, distribution and reproduction in any medium or format, as long as you give appropriate credit to the original author(s) and the source, provide a link to the Creative Commons license, and indicate if changes were made. The images or other third party material in this article are included in the article's Creative Commons license, unless indicated otherwise in a credit line to the material. If material is not included in the article's Creative Commons license and your intended use is not permitted by statutory regulation or exceeds the permitted use, you will need to obtain permission directly from the copyright holder. To view a copy of this license, visit http://creativecommons.org/licenses/by/4.0/
